# FHR-5 Serum Levels and *CFHR5* Genetic Variations in Patients With Immune Complex-Mediated Membranoproliferative Glomerulonephritis and C3-Glomerulopathy

**DOI:** 10.3389/fimmu.2021.720183

**Published:** 2021-09-10

**Authors:** Nóra Garam, Marcell Cserhalmi, Zoltán Prohászka, Ágnes Szilágyi, Nóra Veszeli, Edina Szabó, Barbara Uzonyi, Attila Iliás, Christof Aigner, Alice Schmidt, Martina Gaggl, Gere Sunder-Plassmann, Dóra Bajcsi, Jürgen Brunner, Alexandra Dumfarth, Daniel Cejka, Stefan Flaschberger, Hana Flögelova, Ágnes Haris, Ágnes Hartmann, Andreas Heilos, Thomas Mueller, Krisztina Rusai, Klaus Arbeiter, Johannes Hofer, Dániel Jakab, Mária Sinkó, Erika Szigeti, Csaba Bereczki, Viktor Janko, Kata Kelen, György S. Reusz, Attila J. Szabó, Nóra Klenk, Krisztina Kóbor, Nika Kojc, Maarten Knechtelsdorfer, Mario Laganovic, Adrian Catalin Lungu, Anamarija Meglic, Rina Rus, Tanja Kersnik Levart, Ernesta Macioniene, Marius Miglinas, Anna Pawłowska, Tomasz Stompór, Ludmila Podracka, Michael Rudnicki, Gert Mayer, Romana Rysava, Jana Reiterova, Marijan Saraga, Tomáš Seeman, Jakub Zieg, Eva Sládková, Natasa Stajic, Tamás Szabó, Andrei Capitanescu, Simona Stancu, Miroslav Tisljar, Kresimir Galesic, András Tislér, Inga Vainumäe, Martin Windpessl, Tomas Zaoral, Galia Zlatanova, Mihály Józsi, Dorottya Csuka

**Affiliations:** ^1^Department of Internal Medicine and Haematology, Semmelweis University, Budapest, Hungary; ^2^MTA-ELTE Complement Research Group, Eötvös Loránd Research Network (ELKH), Department of Immunology, ELTE Eötvös Loránd University, Budapest, Hungary; ^3^Research Group for Immunology and Haematology, Semmelweis University-Eötvös Loránd Research Network (Office for Supported Research Groups), Budapest, Hungary; ^4^Department of Immunology, ELTE Eötvös Loránd University, Budapest, Hungary; ^5^Division of Nephrology and Dialysis, Department of Medicine III, Medical University of Vienna, Vienna, Austria; ^6^1st Department of Internal Medicine, University of Szeged, Szeged, Hungary; ^7^Department of Pediatrics, Medical University of Innsbruck, Innsbruck, Austria; ^8^Department of Medicine III: Nephrology, Transplant Medicine and Rheumatology, Geriatric Department, Ordensklinikum Linz-Elisabethinen, Linz, Austria; ^9^Hospital of Klagenfurt, Klagenfurt, Austria; ^10^Division of Nephrology, Department of Pediatrics, Faculty of Medicine, Palacky University and Faculty Hospital in Olomouc, Olomouc, Czechia; ^11^Department of Nephrology, Péterfy Hospital, Budapest, Hungary; ^12^Department of Pediatrics, University of Pécs, Pécs, Hungary; ^13^Department of Pediatrics and Adolescent Medicine, Division of Pediatric Nephrology and Gastroenterology, Medical University of Vienna, Vienna, Austria; ^14^Institute of Neurology of Senses and Language, Hospital of St John of God, Linz, Austria; ^15^Research Institute for Developmental Medicine, Johannes Kepler University Linz, Linz, Austria; ^16^Department of Pediatrics, University of Szeged, Szeged, Hungary; ^17^Medimpax, Bratislava, Slovakia; ^18^1st Department of Pediatrics, Semmelweis University, Budapest, Hungary; ^19^Fresenius Medical Care (FMC) Center of Dialysis, Miskolc, Hungary; ^20^Institute of Pathology, Faculty of Medicine, University of Ljubljana, Ljubljana, Slovenia; ^21^Department of Nephrology, Wilhelminenspital, Vienna, Austria; ^22^Department of Nephrology, Arterial Hypertension, Dialysis and Transplantation, University Hospital Center Zagreb, School of Medicine, University of Zagreb, Zagreb, Croatia; ^23^Pediatric Nephrology Department, Fundeni Clinical Institute, Bucharest, Romania; ^24^Department of Pediatric Nephrology, Division of Pediatrics, University Medical Center Ljubljana, Ljubljana, Slovenia; ^25^Nephrology Center, Santaros Klinikos, Medical Faculty, Vilnius University, Vilnius, Lithuania; ^26^Department of Nephrology, Hypertension and Internal Medicine, School of Medicine, Collegium Medicum, University of Warmia and Mazury, Olsztyn, Poland; ^27^Department of Pediatrics, Comenius University, Bratislava, Slovakia; ^28^Department of Internal Medicine IV-Nephrology and Hypertension, Medical University Innsbruck, Innsbruck, Austria; ^29^Nephrology Clinic, 1st Faculty of Medicine, Charles University, Prague, Czechia; ^30^Department of Pediatrics, University Hospital Split, Split, Croatia; ^31^School of Medicine, University of Split, Split, Croatia; ^32^Department of Pediatrics, 2nd Faculty of Medicine, Charles University Prague, University Hospital Motol, Pragu, Czechia; ^33^Department of Pediatrics, Faculty of Medicine in Pilsen, Charles University in Prague, Pilsen, Czechia; ^34^Institute of Mother and Childhealth Care of Serbia “Dr Vukan Čupić”, Belgrade, Serbia; ^35^Department of Pediatrics, Faculty of Medicine, Debrecen University, Debrecen, Hungary; ^36^Carol Davila Nephrology Hospital, Bucharest, Romania; ^37^Department of Nephrology, University Hospital Dubrava Zagreb, Zagreb, Croatia; ^38^Department of Internal Medicine and Oncology, Semmelweis University, Budapest, Hungary; ^39^Department of Pathology, Tartu University Hospital, Tartu, Estonia; ^40^Internal Medicine IV, Section of Nephrology, Klinikum Wels-Grieskirchen, Wels, Austria; ^41^Department of Pediatrics, University Hospital and Faculty of Medicine, Ostrava, Czechia; ^42^University Children’s Hospital, Medical University, Sofia, Bulgaria

**Keywords:** membranoproliferative glomerulonephritis, immune complex-mediated glomerulonephritis, C3 glomerulopathy, dense deposit disease (DDD), C3 glomerulonephritis (C3GN)

## Abstract

**Background:**

Factor H-related protein 5 (FHR-5) is a member of the complement Factor H protein family. Due to the homology to Factor H, the main complement regulator of the alternative pathway, it may also be implicated in the pathomechanism of kidney diseases where Factor H and alternative pathway dysregulation play a role. Here, we report the first observational study on *CFHR5* variations along with serum FHR-5 levels in immune complex-mediated membranoproliferative glomerulonephritis (IC-MPGN) and C3 glomerulopathy (C3G) patients together with the clinical, genetic, complement, and follow-up data.

**Methods:**

A total of 120 patients with a histologically proven diagnosis of IC-MPGN/C3G were enrolled in the study. FHR-5 serum levels were measured in ELISA, the *CFHR5* gene was analyzed by Sanger sequencing, and selected variants were studied as recombinant proteins in ELISA and surface plasmon resonance (SPR).

**Results:**

Eight exonic *CFHR5* variations in 14 patients (12.6%) were observed. Serum FHR-5 levels were lower in patients compared to controls. Low serum FHR-5 concentration at presentation associated with better renal survival during the follow-up period; furthermore, it showed clear association with signs of complement overactivation and clinically meaningful clusters.

**Conclusions:**

Our observations raise the possibility that the FHR-5 protein plays a fine-tuning role in the pathogenesis of IC-MPGN/C3G.

## Introduction

Membranoproliferative glomerulonephritis (MPGN) is a well-described histological pattern on light microscopy characterized by the pathological presence of capillary wall thickening with double-contour formation, mesangial hypercellularity, and endocapillary proliferation on kidney biopsies (1). Current classification divides MPGN into complement-mediated C3 glomerulopathy (C3G) and immune complex-mediated membranoproliferative glomerulonephritis (IC-MPGN) based on the immunofluorescence microscopy findings where C3 staining is minimum two-order magnitude stronger than any other immunoreactant in the case of C3G (2). C3G is further divided into C3 glomerulonephritis (C3GN) and dense deposit disease (DDD). C3GN is characterized by less dense mesangial, paramesangial, subendothelial, and subepithelial deposits, whereas DDD is characterized by dense intramembranous deposits (2). According to current classification, the dysregulation of the alternative pathway (AP) of complement system is responsible for C3G, which may be caused by autoantibodies against different complement components [e.g., C3 nephritic factor (C3NeF), C4 nephritic factor (C4NeF), C5 nephritic factor (C5NeF) (3), anti-Factor H, anti-C3 (4–8)], anti-Factor B (9), and/or (rare) variations in complement-associated genes (*C3*, *CFH*, *CFI*, *CFB*, *THBD*, *CD46*) (10–12). Interestingly, the abovementioned pathologic factors can be detected in only 30%–80% of the patients (1, 5, 10–15). However, in many cases, it may be difficult to distinguish between IC-MPGN and C3G or C3GN and DDD. Signs of AP dysregulation, such as decreased C3 or the presence of C3NeF, can be detected in IC-MPGN as well, and repeated biopsies may show changes in histology patterns (2, 16–18). For these reasons, we included both entities in our study. A novel, hypothesis-free, data-driven analysis of MPGN patients raised the possibility that clinically meaningful clusters may replace the histology-based classification (19). Furthermore, there are many cases with signs of AP dysregulation without any well-known pathogenic factor (14), but there are new candidates that may broaden the group of possible pathogenic factors. One potential candidate is complement Factor H (FH)-related protein 5 (FHR-5), which was identified in 2001 (20). FHR-5 is a member of the complement FH protein family (FHR), which consists of the AP complement inhibitors FH and FH-like 1, which are derived by alternative splicing from *CFH*, and five FH-related (FHR) proteins that are highly homologous to FH but lack the domains responsible for the complement regulatory activity of FH, and their functions are not fully understood (21). This homology raises the possibility that FHRs can compete with FH for the binding of C3b (22, 23). Moreover, FHR-5 can bind to heparin, C-reactive protein, pentraxin-3, and the extracellular matrix (22, 24), and it can also inhibit the C3- and C5-convertases based on previous studies (22, 25, 26). Mutant FHR-5 was described as a pathogenic factor in a subtype of C3G (27–29). This hereditary endemic form in Cyprus was named CFHR5-nephropathy, which is caused by an internal duplication of exons 2 and 3 of the *CFHR5* gene (28). This entity presents with synpharyngitic macroscopic hematuria with renal failure (30). However, this endemic form is caused by a special duplicated form of the FHR-5 protein, which is characterized by an altered function compared to the wild-type protein. All these facts turned the researchers’ attention on further possible functions of this protein that may explain the molecular background or its association with diseases. In recent years, genetic analysis of *CFHR5* in several conditions such as atypical hemolytic uremic syndrome (aHUS), IgA-nephropathy (IgAN), or MPGN explored a potential connection with these pathological states (27, 31–33), but the exact pathophysiological role of FHR-5 is still unknown. Large case series studies that examined the patients’ FHR-5 levels have been performed only in patients with IgAN (34, 35). Interestingly, FHR-5 serum levels were elevated in patients with IgAN and showed an association with disease progression (34, 35). In a small cohort of 23 C3GN patients, lower FHR-5 levels were detected compared to healthy controls (36). Moreover, genetic variations of *CFHR5* were described in a cohort of 104 C3G patients, but its pathological role is unclear (37). Remarkably, worldwide databases publish different frequencies of *CFHR5* variations in the population. Probably not only the FHR proteins’ plasma levels but the modified FHR and FH plasma repertoire may also affect the complement regulation on the endothelial surface or in the fluid-phase resulting in cell injury. Interactions with glycosaminoglycans can also affect the functions of the abovementioned proteins (26). However, detailed functional and genetic analysis of *CFHR5* along with the parallel measurement of serum levels of the protein in comparison with patients’ clinical, complement, and genetic data has not been performed in a large number of IC-MPGN and C3G patients.

Our aim was to analyze the FHR-5 protein serum levels in a large group of IC-MPGN and C3G patients to better understand the possible role of the FHR-5 protein in disease pathogenesis or disease course and to screen the sequence of *CFHR5*. We also explored the connection of FHR-5 levels and *CFHR5* variations with the recently described clinically relevant clusters in MPGN (4, 19, 38).

## Materials and Methods

### Patients and Control Subjects

Samples of 206 patients were sent to our research laboratory from Central-European clinical centers (n = 34) with the suspicion of complement-mediated renal disease for complement investigations, and genetic analysis was also carried out. Among them, 86 patients were excluded because of alternative diagnosis or secondary MPGN. One hundred twenty patients with the diagnosis of IC-MPGN/C3G were enrolled in the study from January 2008 to May 2019.

The clinical, histological, and laboratory data were collected from the clinicians and pathologists according to study protocols approved by the Medical Research Council of the Ministry of Human Capacities in Hungary (approval number: 55381-1/2015/EKU) and the institutional review board of the Semmelweis University, Budapest.

Biopsy data were collected using standardized questionnaire forms from pathologists (n = 73) or extracted from the biopsy descriptions (n = 47).

Eighty-five subjects formed the control group (68 adults, 17 children). All of them were referred for routine medical examination, and none of them had any known disease at the time of blood sampling. **Informed consent** was obtained from all healthy controls and patients, or, if patients were under 18 years of age, from a parent and/or legal guardian. The study was conducted along the lines of the Declaration of Helsinki.

### ELISA for Measuring the Serum Level of FHR-5

**Figure 1:**
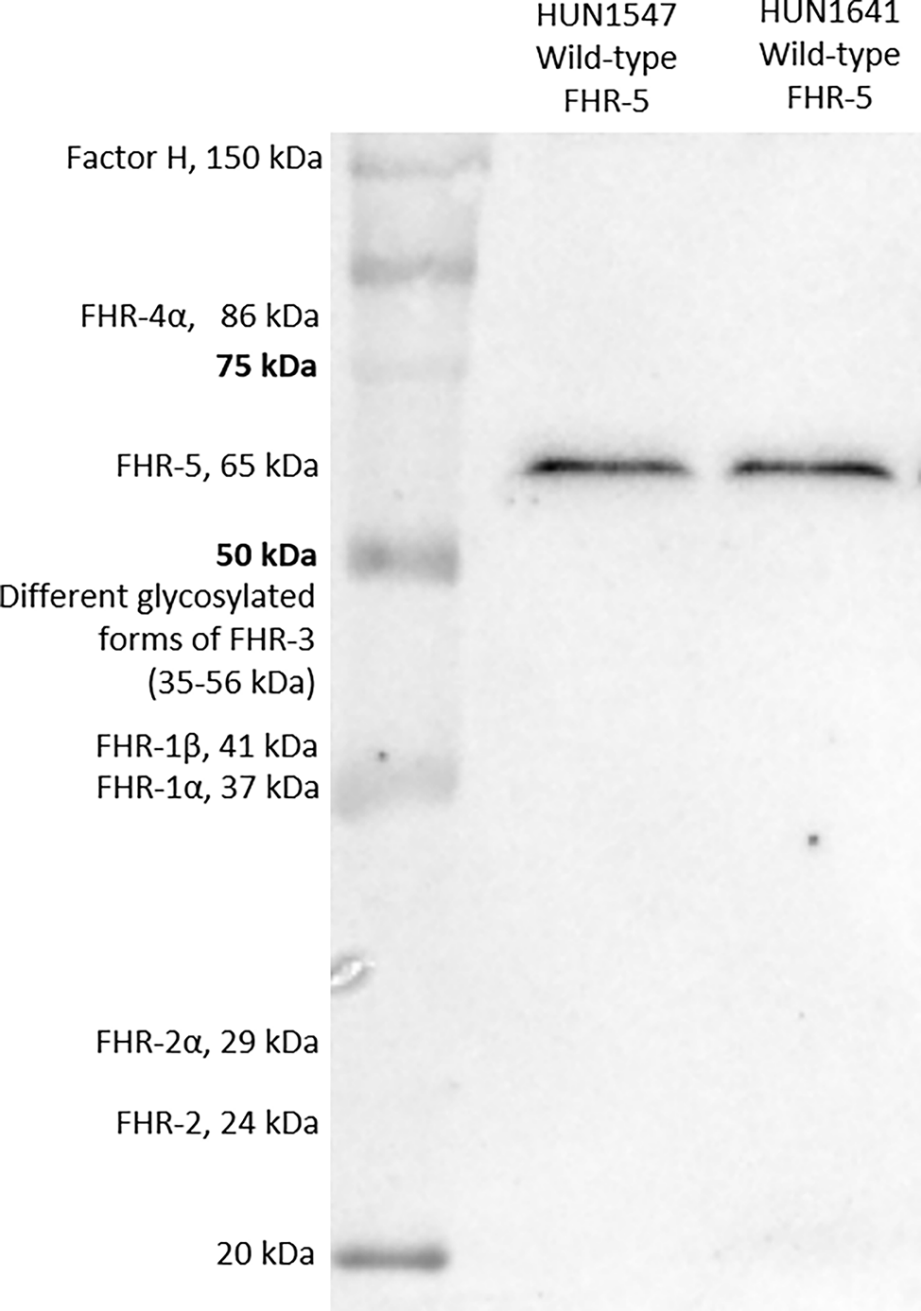
Factor H-related protein 5 (FHR-5) on Western blot. Two serum samples obtained from patients with wild-type FHR-5 protein were analyzed.

FHR-5 serum levels were measured with newly developed in-house ELISA method. Microtiter ELISA plates were coated with 1 μg/ml commercially available monoclonal mouse anti-human FHR-5 (IgG1, clone #390513, R&D Systems, Minneapolis, Minnesota, US) in phosphate-buffered saline (PBS) overnight, followed by blocking with PBS and 2% bovine serum albumin (BSA) the next day. Serum was diluted 1:100 in PBS containing 1% BSA and 0.05% Tween-20 and added to the plate [1 h, room temperature (RT)]. FHR-5 binding was detected using polyclonal goat anti-human FHR-5 IgG (Cat. number: AF3845, R&D Systems, Minneapolis, Minnesota, US). Concentrations of the samples were determined based on the standard curve of 2-fold dilution series of recombinant human FHR-5 protein (R&D Systems, Minneapolis, Minnesota, US). Inter-assay and intra-assay variations were determined as 11.8% and 7.8%, respectively. Specificity of the FHR-5 ELISA was confirmed by Western blot (WB) showing that the monoclonal anti-FHR-5 used as capture antibody did not detect FH or any FHR other than FHR-5 (**Figure 1**).

### Western Blot for Detection of FHR-5

Serum proteins from patients and a healthy individual were separated on 10% sodium dodecyl sulfate–polyacrylamide gel electrophoresis (SDS-PAGE) under non-reducing conditions and blotted onto nitrocellulose membrane. After blotting, the membrane was blocked with 4% non-fat dried milk, 1% BSA in PBS solution. Then, the membrane was incubated with a polyclonal goat anti-FHR-5 (Cat. number: AF3845, R&D Systems, Minneapolis, Minnesota, US) or monoclonal mouse anti-FHR-5 antibody (clone #390513, Cat. number: MAB3845, R&D Systems, Minneapolis, Minnesota, US) followed by horseradish peroxidase (HRP)-conjugated secondary antibodies (rabbit anti-goat and goat anti-mouse, respectively; Southern Biotech, Birmingham, AL, USA). Bound antibodies were detected with Clarity Western ECL substrate (Bio-Rad, Hercules, CA, USA). For estimating the molecular weight of the proteins, a protein molecular weight marker containing a mixture of 10 multicolor recombinant proteins was used (Precision Plus Protein™ Kaleidoscope™ Prestained Protein Standard, Bio-Rad, Hercules, CA, USA).

### Molecular Genetic Analysis

In 111 patients, the whole coding region of the gene encoding FHR-5 (*CFHR5*; OMIM #608593) was screened by direct bidirectional DNA sequencing, as described in the case of disease-associated genes (*CFH*, *CFI*, *CD46*, *THBD*, *CFB*, and *C3*) that were sequenced in the same population previously (39). No DNA samples were available from the remaining nine patients. Sequencing was not performed in the included healthy subjects; minor allele frequencies of variations in larger populations available from public databases (1000Genomes Project, EVS, gnomAD) were used instead. Primer sequences and PCR conditions are available upon request.

Polymorphic variations are numbered +1 from the A allele of the ATG translation initiation site. The possible functional effect of the identified variations was predicted *in silico* using online prediction tools, such as PolyPhen (version2) (40) (http://genetics.bwh.harvard.edu/pph2/), SIFT (41) (http://siftdna.org/www/Extended SIFT chrcoords submit.html), PROVEAN (42) (http://provean.jcvi.org/genomesubmit.php), Human Splicing Finder (version 3.1; http://www.umd.be/HSF3/) (43), and Mutation-Taster (44) (http://mutationtaster.org).

### Generation of Recombinant Wild-Type and Mutant FHR-5 Variants

Coding sequence of wild-type *CFHR5* (wt*CFHR5*) was codon-optimized for insect cell expression system (Integrated DNA Technologies, Inc.) and cloned into the pBSV-8His expression vector (45). Two mutants were amplified from the wt*CFHR5*-containing vector with mutagenic forward primers introducing the mutations FHR-5_G278S_ and FHR-5_R356H_. Sequences and mutations were confirmed by sequencing. Recombinant proteins were produced in *Spodoptera frugiperda* (Sf9) cells after co-transfection of the various *CFHR5*-containing expression vectors with linearized baculovirus DNA (Oxford Expression Technologies Ltd., Oxford, UK) and purified from the supernatant by nickel affinity chromatography.

### Measurement of the Interaction of FHR-5 With Purified C3b

To compare the C3b-binding ability of FHR-5_WT_, FHR-5_G278S_, and FHR-5_R356H_, microtiter plate wells (MaxiSorp, Nunc, Thermo Fisher Scientific, Waltham, MA, USA) were coated with 5 µg/ml C3b (Merck, Darmstadt, Germany). After blocking with 5% BSA in 0.05% Tween-20 containing Dulbecco’s phosphate buffer saline (DPBS) (Lonza, Basel, Switzerland), serial dilutions of recombinant FHR-5 variants were added to the wells for 1 h at 20°C. Binding was detected with polyclonal goat anti-human FHR-5 IgG (Cat. number: AF3845, R&D Systems, Minneapolis, Minnesota, US) followed by HRP-labeled rabbit anti-goat Ig (Dako, Hamburg, Germany). The binding was visualized using 3,3′,5,5′-tetramethylbenzidine (TMB) (BioLegend, San Diego, CA, US), and the absorbance was measured at 450/620 nm.

The interaction of the recombinant FHR-5 variants with C3b was also analyzed by surface plasmon resonance (SPR) method using ProteOn XPR36 equipment (Bio-Rad, Hercules, CA, USA). Recombinant proteins and ovalbumin (Sigma-Aldrich, St. Louis, Missouri, US) as negative control were diluted in 10 mM Na-acetate buffer (pH 4.0) and immobilized vertically at a density of 4,200–4,600 RU on GLC biosensor chip by standard amine coupling method. As analyte, serial dilutions of C3b were prepared in DPBS containing 0.005% Tween-20 and injected in the horizontal orientation of channels over immobilized recombinant FHR-5 variants. Measurements were performed at 50 μl/min flow rate; association was followed for 120 s and the dissociation for 600 s. Data were processed and analyzed with ProteOnManager software. The curves were corrected by subtracting the nonspecific binding responses obtained from control, the ovalbumin captured channel. Binding curves were fit to bivalent analyte model, and the equilibrium dissociation constants were calculated from the directly estimated association and dissociation rate constants (K_D_ = k_d_/k_a_). The experiment was performed twice on separate GLC biosensor chips.

To measure the C3b-binding ability of the native (wild-type or mutant) FHR-5 from serum samples, microtiter plate wells (MaxiSorp, Nunc, Thermo Fisher Scientific, Waltham, MA, USA) were coated with 5 µg/ml C3b fragment (Merck, Darmstadt, Germany) [overnight (ON), 4°C]. After blocking with DPBS containing 2% BSA (1 h, RT), serum samples diluted 1:4 in DPBS containing 1% BSA, 0.05% Tween 20, and a dilution series (3.9–250 ng/ml) of recombinant human FHR-5 (R&D Systems, Minneapolis, Minnesota, US) were applied to the plate (1 h, 37°C). Binding was detected with monoclonal mouse anti-human FHR-5 (R&D Systems, Minneapolis, Minnesota, US) (1 h, RT), followed by HRP-labeled goat anti-mouse IgG (Jackson ImmunoResearch, Ely, UK) (1 h, RT). Substrate TMB (BioLegend, San Diego, CA, US) was used, and the absorbance was measured at 450/620 nm.

### Determinations of Complement Parameters

Samples [serum, ethylenediaminetetraacetic acid (EDTA)-anticoagulated plasma, and sodium citrate-anticoagulated plasma] were taken from the antecubital vein or from a central venous catheter. Cells and supernatants were separated by centrifugation after the sample was taken and transferred to our laboratory. Separated aliquots were stored at −70°C until measurements.

C3, C4 concentrations were measured by turbidimetry (Beckman Coulter, Brea, CA, USA).

AP activation was measured by a commercially available kit (Wieslab AP ELISA KITs, EuroDiagnostica, Malmö, Sweden) according to the manufacturer’s instructions.

Total CP activity was measured by a home-made hemolytic titration test based on Mayer’s method (46). Radial immunodiffusion was performed to measure the antigenic concentrations of Factor I and Factor B using specific antibodies (47). Levels of FH, C1q, and antibodies against FH, C1q (47–49), C3, and Factor B were measured with in-house ELISA methods (4), whereas C3NeF and C4NeF titer was determined based on hemolytic method (4, 50).

Further complement components, activation markers, and split products, such as Factor D, sC5b-9, C3a, Bb, and C4d were detected with commercially available ELISA kits (Hycult, Uden, The Netherlands, Complement Factor D, Human, ELISA kitHK343-02; MicroVue C3a-desArgEIA, A032; MicroVue C4d EIA, A008; MicroVue, Quidel, San Diego, CA, USA sC5b-9 Plus EIA, A029; MicroVue Bb Plus EIA, A027, respectively).

### Statistical Analysis

For descriptive purposes, continuous variables that were deviated from the normal distribution according to the results of Shapiro–Wilk tests are given as medians and 25th–75th percentiles. For categorical variables, numbers and percentages were used. Nonparametric tests as Mann–Whitney U test or Kruskal–Wallis test with Dunn’s *post-hoc* test were used for group comparisons in case of continuous variables. For categorical variables, Pearson’s χ^2^ test was performed. For comparison of the recombinant FHR-5 variants’ data, repeated-measures ANOVA was used with Tukey’s multiple comparison test. Correlation r –value and significance levels were determined using Spearman correlations test.

For cluster analysis, hierarchical clustering by Ward method with squared Euclidean distances was used, as described previously (38).

In order to split FHR-5 levels into high and low groups, receiver operating characteristic (ROC) analysis was made. Kaplan–Meier analysis with log-rank test was performed to examine patients’ renal survival.

For the statistical analysis, IBM SPSS Statistics 20 and GraphPad Prism 5 software were used. Two-tailed p-values were calculated, and the significance level was determined at a value of p < 0.05 if not otherwise stated.

## Results

### Baseline Characteristics of the Patients

One hundred twenty patients with a diagnosis of IC-MPGN/C3G and 85 healthy individuals were enrolled in the study. Forty-one patients had C3GN, 12 had DDD, and 67 had IC-MPGN. Detailed descriptive characteristics of the patients were reported previously (4). There was no difference between patients diagnosed with MPGN and healthy control subjects with regard to gender and age distribution. However, C3, C4, AP, and classical pathway (CP) activity were decreased in patients with IC-MPGN/C3G compared to those in healthy subjects (**Table 1**). There was no difference between the different histology-based groups regarding clinical characteristics such as proteinuria (p = 0.2), hematuria (p = 0.85), renal impairment/failure (p = 0.84), triggering event (p = 0.55), or familiarity (p = 0.23). Based on our database, most of the patients received antihypertensive drugs [angiotensin-converting enzyme (ACE) inhibitors, angiotensin II receptor blockers] and immunosuppressants (steroid, cyclophosphamide, mycophenolate mofetil). Furthermore, plasma therapy and renal replacement therapy were indicated at the time of diagnosis in some cases.

**Table 1:** Clinical characteristics of patients with C3G/IC-MPGN.

	C3GN (n = 41)	DDD (n = 12)	IC-MPGN (n = 67)	Total (n = 120)	Healthy control (n = 85)		p*
Sex % men	22 (53.6)	3 (25)	43 (64.2)	68 (56.6)	39 (45.9)		0.156
Age at diagnosis, years	22 (15–38)	22 (16–42)	19 (11–41)	22 (13–40)	31 (25–36)		0.06
*Clinical data*							
Non-visible hematuria, present	25 (61)	8 (66.6)	38 (56.7)	71(61.2)			
Visible hematuria, present	9 (22)	2 (16.6)	12 (18)	23 (19.8)			
Nephrotic syndrome, present	19 (46.3)	9 (75)	33 (29.3)	61 (52.1)			
Renal impairment, present	14 (34.1)	5 (41.6)	26 (38.8)	45 (38.4)			
Renal failure, present	5 (12.2)	1 (8.3)	6 (8.9)	12 (10.2)			
Trigger, present	8 (19.5)	3 (25)	10 (15)	21 (17.5)			
Familiarity, present	6 (14.6)	0 (0)	5 (7.5)	11 (9.1)			
*Complement data*							
Serum C3, g/L	0.68 (0.27–1.06)	0.49 (0.25–0.87)	0.7 (0.48–0.99)	0.69 (0.3–1)		1.2 (1.1–1.4)	**<0.0001**
Serum C4, g/L	0.28 (0.2–0.39)	0.21 (0.16–0.37)	0.21 (0.12–0.25)	0.22 (0.16–0.31)		0.32 (0.27–0.37)	**<0.0001**
Classical pathway activity, CH50/ml	38 (19–62)	47 (23–57)	46 (30–60)	46 (26–60)		64 (56–72)	**<0.0001**
Alternative pathway activity (Alt), %	38 (1–78)	4 (0.3–66)^1^	70 (13–95)	58 (1–86)		91 (77–104)	**<0.0001**
Decreased C3	21 (51.2)	10 (83.8)	37 (55.2)	68 (56.6)		0 (0)	**<0.0001**
Decreased C3 with normal C4	18 (43.9)	10 (83.8)	25 (37.3)	53 (44.2)		0 (0)	**<0.0001**
Serum FHR-5, mg/L	1.8 (1.5–2.6)	1.6 (1.4–2)	1.8 (1.3–2.2)	1.8 (1.4–2.3)		2.1 (1.8–2.5)	**0.004**
sC5b-9, ng/ml	429 (277–809)	453 (248–970)	376 (248–658)	407 (256–719)			
Elevated sC5b-9	28 (75.6)	7 (77.7)	45 (75)	80 (75.4)			
C1q, mg/L	108 (90–130)	95 (83–107)	101 (69–123)	104 (83–123.75)			
Factor H, mg/L	528 (470–697)	715 (589–903)	495 (324–700)	534 (381–715)			
Factor I, %	93 (79–112)	87 (78–98)	90 (74–110)	91 (78–109)			
Factor B, %	90 (72–106)	88 (69–124)	91 (72–106)	86 (67–103)			
Factor D, µg/ml	1.9 (0.7–4.4)	2.8 (0.8–4)	2.4 (0.95–3.6)	2.31 (0.9–3.94)			
C3a, ng/ml	160 (70–259)	221 (59–259)	125 (86–183)	132 (79–208)			
Bb, µg/ml	1.57 (1.12–2.6)	1.7 (0.05–3.7)	1.4 (0.9–2)	1.49 (0.99–2.28)			
C4d, ng/ml	6.2 (2.9–8.8)	6.1 (3.3–9.4)	4.1 (3–8.8)	5.19 (3.1–8.9)			
C3NeF, present	7 (17.1)	5 (41.6)	15 (22.4)	27 (22.5)			
C4NeF, present	7 (17.5)	1 (8.3)	9 (13.4)	17 (14.2)			
anti-Factor H, present	4 (10)	0 (0)	3 (4.5)	7 (5.9)			
anti-C1q, present	5 (12.8)	1 (8.3)	9 (14.3)	15 (13.4)			
anti-C3, present	2 (5.4)	1 (8.3)	2 (3)	5 (4.3)			
anti-Factor B, present	3 (8.1)	2 (16.6)	2 (3)	7 (6)			
Positivity for >1 complement autoantibody^+^	7 (18.9)	1 (10)	8 (12.7)	16 (15.1)			
LPV carriers**	7 (17.1)	2 (16.6)	13 (19.4)	22 (19.8)			

Data presented are number (%) or median (interquartile range).

FHR-5, Factor H-related protein 5.

*Group comparisons were made with Mann–Whitney U test between “total” and “controls.”

**LPVs were detected in the following genes: CFH, CFI, CFB, C3, CD46, THBD.

^+^The analyzed autoantibodies: anti-Factor H, anti-C3, anti-Factor B, C3NeF, C4NeF

Reference ranges: C1q: 60–180 mg/L; C3: 0.9–1.8 g/L; C4: 0.15–0.55 g/L; CH50: 48–103 CH50/ml; Alt: 70%–105%; Bb: 0.49–1.42 μg/ml; C4d: 0.7–6.3 μg/ml; sC5b-9: 110–252 ng/ml; Factor D: 0.51–1.59 μg/ml; Factor H: 250–880 mg/L; Factor I: 70%–130%; Factor B: 70%–130%.

C3G, C3 glomerulopathy; DDD, dense deposit disease; IC-MPGN, immune complex-mediated membranoproliferative glomerulonephritis, LPV, likely pathogenic variation; C3GN, C3 glomerulonephritis.

p-values < 0.05 are shown in bold.

Complement parameters did not show any significant differences except for C4 levels that were lower in patients with IC-MPGN and AP activity that was the lowest in the DDD group. Serum FHR-5 levels were significantly lower in patients [median: 1.8 mg/L, interquartile range (IQR): 1.4–2.3] compared to healthy controls (median: 2.1 mg/L, IQR: 1.8–2.5) (p = 0.004) (**Table 1**), and there was no difference between the various histology-based groups.

### Analysis of *CFHR5* Variations in Patients With IC-MPGN or C3G

In order to examine whether lower FHR-5 levels in patients can be attributed to variations in the *CFHR5* gene, genetic analysis was performed with Sanger sequencing in 111 patients. Altogether, eight different heterozygous *CFHR5* variations including two frameshift mutations (c.479_480insAA and c.479_480insA) and six missense variations (P46S, V110A, K144N, C208R, G278S, and R356H) were identified in 14 patients. In addition, we have identified three different *CFHR* hybrid genes in our patients that were excluded from further statistical analysis (separate manuscript in preparation for the detailed characterization of the hybrid genes).

We compared the clinical and laboratory data of patients carrying at least one *CFHR5* variation (n = 14 patients with *CFHR5* mutations) to patients without a *CFHR5* genetic variation (n = 94) in order to examine whether there are any differences between the patients’ characteristics, but the parameters did not differ significantly between the two groups except for FHR-5 level (**Table 2**).

**Table 2:** Clinical characteristics of patients with or without *CFHR5* variations.

	Patients with variation in *CFHR5* gene (n = 14)	Patients without variations in *CFHR5* gene (n = 94)	p*
Sex % men	8 (57.1)	52 (55.5)	0.87
Age at diagnosis, years	17 (8–29)	22 (13–40)	0.28
Non-visible hematuria, present	8 (57.1)	55 (58.5)	1.00
Visible hematuria, present	3 (21.4)	190 (20.2)	1.00
Nephrotic syndrome, present	8 (57.1)	45 (47.8)	0.57
Renal impairment, present	6 (42.8)	35 (37.2)	0.77
Renal failure, present	0 (0)	12 (12.76)	0.35
Trigger, present	2 (14.28)	18 (19.1)	1.00
Familiarity, present	2 (14.28)	7 (7.4)	0.38
Serum C3, g/L	0.87 (0.47–0.95)	0.8 (0.45–1.16)	0.61
Serum C4, g/L	0.26 (0.17–0.31)	0.28 (0.20–0.37)	0.27
Classical pathway activity, CH50/ml	45 (25–54)	45 (23–60)	0.70
Alternative pathway activity (Alt), %	39 (10–80)	58 (1–84)	0.93
Decreased C3	10 (71.4)	53 (56.3)	0.38
Decreased C3 with normal C4	5 (35.7)	43 (45.7)	0.48
Serum FHR-5, mg/L	1.54 (0.92–1.92)	1.84 (1.43–2.45)	**0.039**
sC5b-9, ng/ml	463 (371–695)	413 (252–852)	0.57
Elevated sC5b-9	12 (85.7)	65 (69.1)	0.2
C1q, mg/L	95 (66.75-110.5)	103 (83.0-125.5)	0.34
Factor H, mg/L	469 (351–606)	542 (386–757)	0.16
Factor I, %	80 (67–102)	93 (78–111)	0.12
Factor B, %	82 (63–98)	86 (66–104)	0.56
Factor D, µg/ml	1.37 (0.67–2.6)	2.3 (0.94–4.15)	0.12
C3a, ng/ml	188 (604–241)	127 (78–206)	0.63
Bb, µg/ml	1.497 (0.62–2.15)	1.52 (1.02–2.37)	0.37
C4d, ng/ml	3.87 (2.67–6.13)	5.42 (3.07–8.99)	0.41
C3NeF, present	2 (14.28)	23 (24.4)	0.39
C4NeF, present	0 (0)	13 (13.8)	0.13
Anti-Factor H, present	2 (14.28)	5 (34.8)	0.2
Anti-C1q, present	2 (14.28)	12 (12.7)	0.97
Anti-C3, present	0 (0)	5 (5.3)	0.37
Anti-Factor B, present	1 (7.1)	6 (6.3)	0.9
Positivity for >1 complement autoantibody^+^	2 (14.2)	17 (18.08)	0.66
LPV carriers**	1 (7.1)	20 (21.2)	0.26

Data presented are number (%) or median (interquartile range).

*Group comparisons were made with Mann–Whitney U test between “total” and “controls.”

**LPVs were detected in the following genes: CFH, CFI, CFB, C3, CD46, THBD

^+^The analyzed autoantibodies: anti-Factor H, anti-C3, anti-Factor B, C3NeF, C4NeF.

Reference ranges: C1q: 60–180 mg/L; C3: 0.9–1.8 g/L; C4: 0.15–0.55 g/L; CH50: 48–103 CH50/ml; Alt: 70%–105%; Bb: 0.49–1.42 μg/ml; C4d: 0.7–6.3 μg/ml; sC5b-9: 110–252 ng/ml; Factor D: 0.51–1.59 μg/ml; Factor H: 250–880 mg/L; Factor I: 70%–130%; Factor B: 70%–130%.

FHR-5, Factor H-related protein 5.

P value in bold refers to a statistically significant difference.

The detailed list of the identified *CFHR5* variations is presented in **Table 3**. Minor allele frequencies of the variations available in different public databases are presented in **Supplementary Table 1**, while *in silico* predictions of their potential functional effect are presented in **Supplementary Table 2**.

**Table 3:** Identified variations in the *CFHR5* gene in patients (n=14) with C3G/IC-MPGN.

Patients’ ID	Diagnosis based on biopsy	Aminoacid change in FHR-5	Variations in other genes	*CFH* Y402H	C3, g/l	C4, g/l	CH50/mL	Alt, %	Factor H, mg/L	sC5b-9, ng/ml	FHR-5, mg/L	Nephrotic syndrome	Renal impairment	Autoantibody
**HUN1502**	IC-MPGN	P46S^(27, 33,51)^	*CD46* T383I^(52-54)^	het.	1.28	0.19	52	65	546	261	1.61	yes	yes	no
**HUN278**	DDD	V110A ^(37,55)^	–	het.	0.87	0.26	46	63	676	383	2.01	yes	yes	C3NeF
**HUN523**	IC-MPGN	V110A ^(37,55)^	*CFI* R406H^(56-59)^	het.	0.93	0.3	38	38	165	534	1.59	no	no	no
**HUN746**	IC-MPGN	V110A ^(37,55)^	*CFI* R406H^(56-59)^ *C3* D277E	wt	0.91	0.5	69	104	732	–	2.03	yes	no	no
**HUN1612**	IC-MPGN	V110A ^(37,55)^	*-*	het	0.45	0.1	30	13	413	731	2.34	yes	yes	no
**HUN821**	IC-MPGN	K144N ^(31,32,37)^	*C3* c.-2_-1insAC	wt	0.19	0	0	0	538	–	1.14	yes	no	anti-C1q
**HUN564**	IC-MPGN	p.E163Kfs*10*	*CD46* A353V^(53,60-63)^	wt	1.02	0.3	0	0	217	165	0.95	no	yes	anti-FH
**HUN593**	IC-MPGN	p.E163Rfs*35 ^(32,36)^	*-*	het	0.41	0.21	35	16	355	368	1.88	no	no	anti-FB
		C208R*												
**HUN2446**	C3GN	P46S^(27, 33,51)^	–	wt	0.24	0.22	12	1	501	1777	0.47	no	no	anti-FH, anti-C1q
		p.E163Rfs*35 ^(32,36)^												
		C208R*												
**HUN225**	DDD	G278S^(37,56,64,65)^	*-*	wt	0.28	0.4	47	7	733	770	0.86	yes	no	no
**HUN769**	IC-MPGN	G278S^(37,56,64,65)^	–	na	0.87	0.36	48	104	583	393	1.9	yes	yes	no
**HUN1190**	IC-MPGN	G278S^(37,56,64,65)^	–	het	0.69	0.12	34	39	379	561	0.62	yes	yes	no
**HUN290**	IC-MPGN	R356H ^(32,66)^	*CD46* G5D*	wt	0.87	0.26	61	87	341	589	1.4	yes	no	C3NeF
**HUN1325**	C3GN	R356H ^(32,66)^	–	wt	1.24	0.28	72	73	438	382	1.5	yes	no	no
**Total, median (interquartile range)**					0.87 (0.42-0.93-	0.26 (0.2-0.3)	42 (31-51)	39 (9-71)	470(361-574)	464(379-625)	1.55(1-1.9)	-	-	-

Reference ranges: C3: 0.9-1.8g/L; C4: 0.15-0.55g/L; CH50: 48-103 CH50/ml; AP: 70-105%; sC5b-9: 110–252ng/mL; Factor H: 250-880 mg/L wt: wild-type; het: heterozygous. CFH Y402H is a common risk factor for dense deposit disease.

Combined mutations occurred in two patients: HUN593 carried both a missense and a frameshift *CFHR5* variation (C208R, c.479_480insA), while HUN2446 carried another missense polymorphism beside the same variations (P46S, C208R, c.479_480insA).

**Figure 2:**
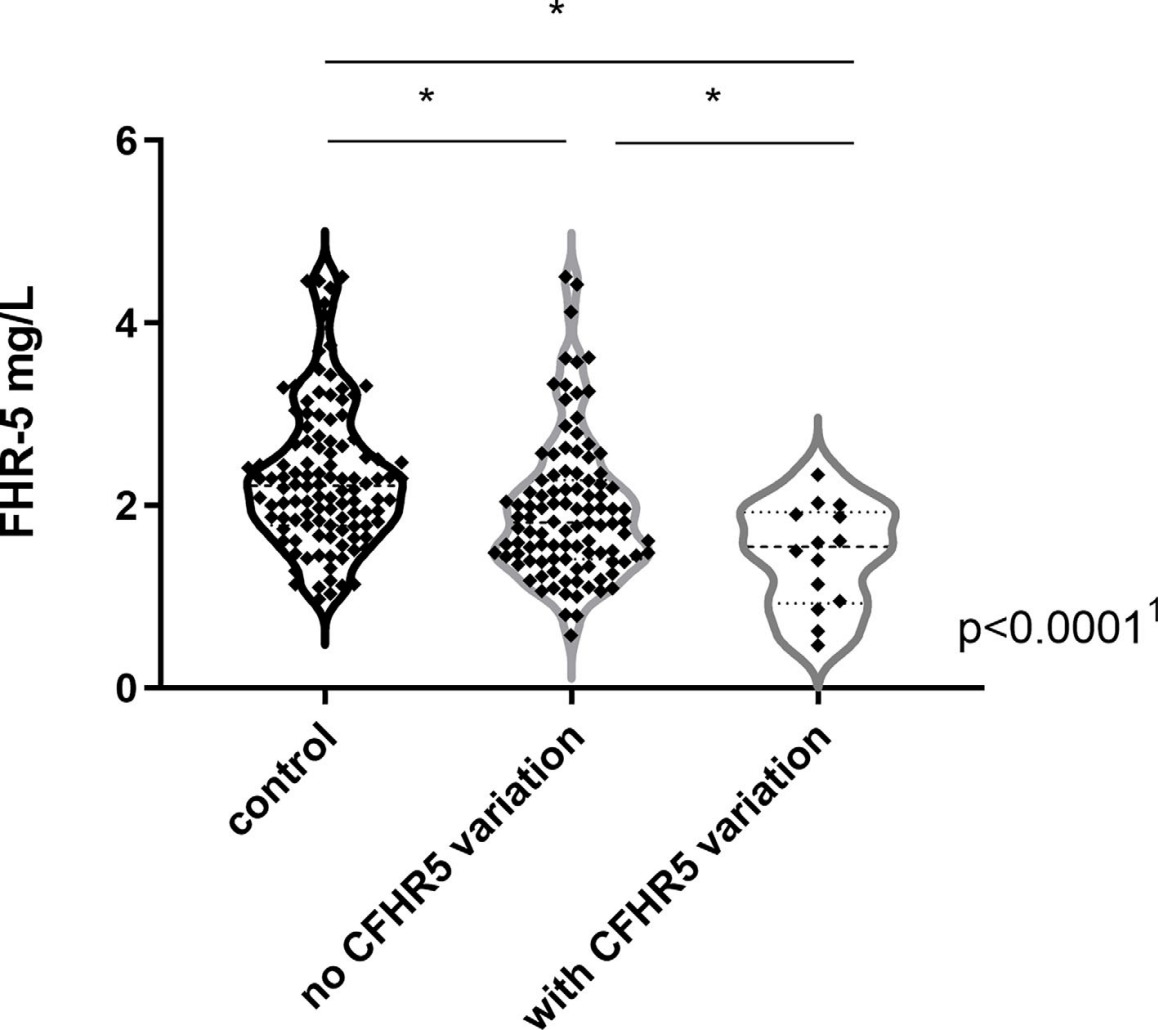
Factor H-related protein 5 (FHR-5) levels in patients with C3 glomerulopathy (C3G)/immune complex-mediated membranoproliferative glomerulonephritis (IC-MPGN) and in healthy individuals. ^1^p-value was determined with Kruskal–Wallis test. *p < 0.05 by Dunn’s posttest.

**Figure 3:**
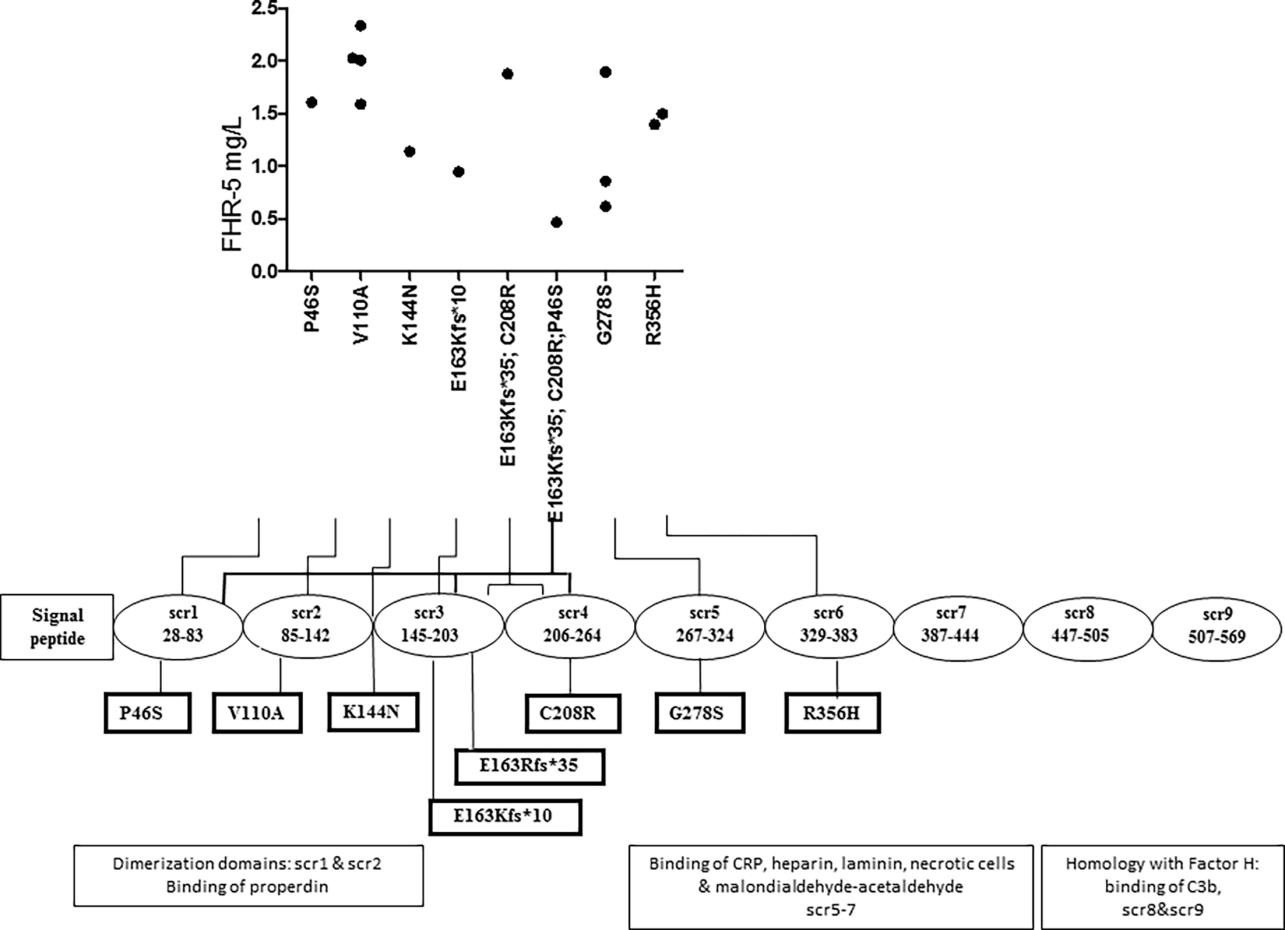
Localization of missense and frame-shift *CFHR5* variations and the serum level of Factor H-related protein 5 (FHR-5).

FHR-5 serum levels were lower in patients compared to controls (median: 2.1 mg/L), regardless of carrying a *CFHR5* variation (median: 1.54 mg/L, p = 0.0004) or without *CFHR5* variations (median: 1.84 mg/L, p = 0.0001) (**Figure 2**). Patients carrying *CFHR5* variations have a lower median FHR-5 level compared to patients with the wild-type protein (p = 0.039). There was no remarkable association of the FHR-5 protein concentrations with the localization of the variations in various FHR-5 domains (**Figure 3**). In patient HUN593 carrying both a missense and a frameshift *CFHR5* variation (C208R, c.479_480insA), the serum level of FHR-5 was not markedly decreased (1.88 mg/L); therefore, this sample was further investigated on WB, where FHR-5 protein was detected in the expected position (**Supplementary Figure 1**) with comparable intensity to that of wild-type FHR-5, confirming the results observed by ELISA (**Figure 3**).

### Functional Characterization of Selected FHR-5 Variants

**Figure 4:**
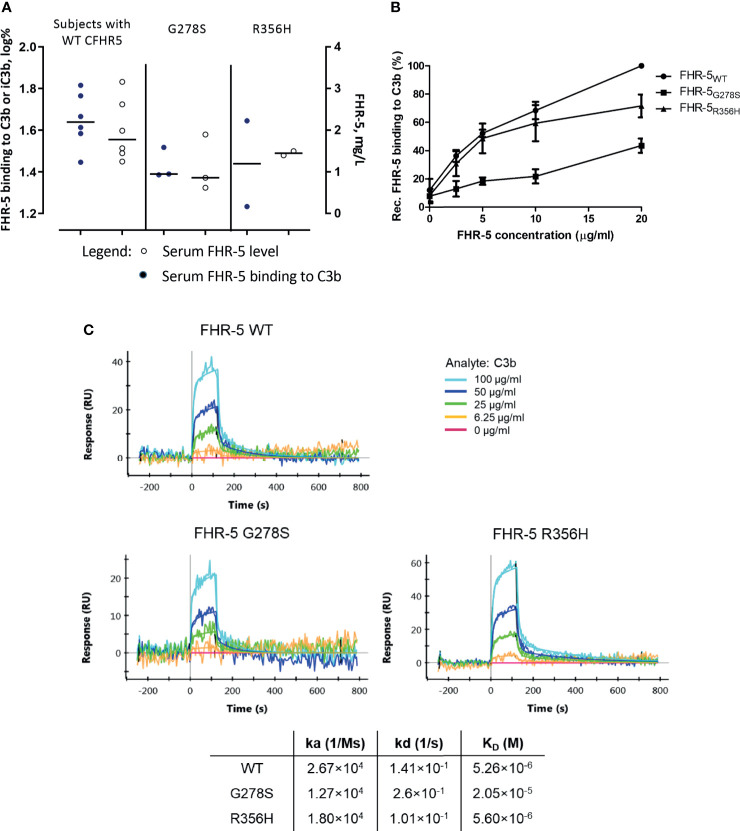
**(A)** Comparison of serum Factor H-related protein 5 (FHR-5) levels and serum FHR-5 C3b-binding ability of patients with only FHR-5_WT_ and those carrying FHR-5_G278S_ or FHR-5_R356H_ in ELISA. C3b was immobilized, then serum was added, and FHR-5 was detected by polyclonal anti-FHR-5. Dots represent individual patients. Data are mean of two measurements and are normalized to a calibrator sample containing only FHR-5_WT_. Subjects expressing FHR-5G278S show a tendency of lower FHR-5 levels and decreased C3b binding compared to the FHR-5WT-expressing group. Serum FHR-5 levels were measured in a sandwich ELISA. Circles represent individual patients. Data are mean of two measurements. **(B)** Dose-dependent binding of recombinant FHR-5_WT_, FHR-5_G278S_, and FHR-5_R356H_ to purified C3b measured in ELISA. The FHR-5_G278S_ variant binds significantly weaker to C3b (one-way ANOVA with Bonferroni’s posttest, p < 0.01). Data are mean of four measurements ± SEM. **(C)** Interaction of C3b–FHR-5 variants measured by surface plasmon resonance (SPR). C3b in serial dilutions was flown over immobilized FHR-5 variants. The K_D_ of the C3b–FHR-5_G278S_ is one order greater compared to that of C3b–FHR-5_R356H_ and C3b–FHR-5_WT_. Data are representative of two experiments.

To determine whether *CFHR5* variations have any influence on FHR-5 functions, we measured native FHR-5 binding to C3b from patients’ sera and selected *CFHR5* variants were analyzed as recombinant proteins. To this end, wild-type FHR-5 (FHR-5_WT_) and two mutants, FHR-5_G278S_ harboring serine instead of glycine at position 278 and FHR-5_R356H_ harboring histidine instead of arginine at position 356, were recombinantly expressed. We chose these two *CFHR5* variations because these were the most abundant *CFHR5* alterations identified in our patients suffering from IC-MPGN or C3G (and atypical hemolytic uremic syndrome). Serum samples of patients carrying the G278S variant showed a tendency of lower C3b binding compared to the patients with wild-type FHR-5 based on ELISA measurements (**Figure 4A**). In ELISA measurements, all three recombinant proteins bound dose-dependently to C3b, FHR-5_G278S_ showing significantly weaker binding (**Figure 4B**). This interaction was also analyzed by SPR method. The K_D_ values calculated for the C3b–FHR-5 interactions are in line with the ELISA results (2.05 × 10^−5^ M for C3b–FHR-5_G278S_ *vs.* 5.26 × 10^-6^ M and 5.60 × 10^-6^ M for C3b–FHR-5_WT_ and C3b–FHR-5_R356H_), since FHR-5_G278S_ has reduced binding to C3b, while FHR-5_R356H_ binds to C3b with similar affinity as the wild-type protein. In detail, C3b showed slower association with both FHR-5_G278S_ and FHR-5_R356H_ (see decreased K_A_ values compared to that of FHR-5_WT_). The dissociation was faster in case of FHR-5_G278S_ and slower in case of FHR-5_R356H_ compared to that of FHR-5_WT_. These differences in association and dissociation result in a weaker interaction with C3b in case of FHR-5_G278S_ (**Figure 4C**).

### FHR-5 Level and Its Associations With Complement Parameters

Next, we aimed to analyze the potential association of serum FHR-5 levels with different laboratory and clinical parameters. We decided to stratify patients carrying *CFHR5* variations into a separate group in order to have a homogeneous group of MPGN patients with wild-type FHR-5 that facilitates better understanding of the association and relevance of FHR-5 protein levels with clinical features and development of end-stage renal disease (ESRD).

**Figure 5:**
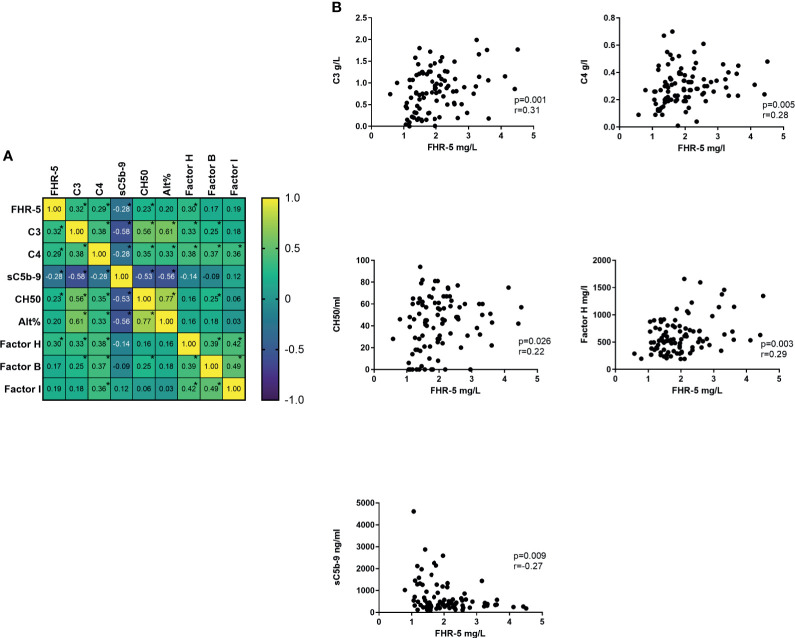
**(A)** Correlation heat map of different complement parameters. Number in boxes indicates Spearman correlation r. *p < 0.05. **(B)** Significant correlation of Factor H-related protein 5 (FHR-5) and other complement parameters.

The FHR-5 serum level showed a positive correlation with the presence of sclerotic glomeruli; however, there was no connection with the presence of hematuria, proteinuria, or any other clinical parameters (data not shown). We have also examined the correlation with the different complement parameters and found significant positive correlations with serum C3, C4, and Factor H levels and the activity of AP and CP. We did not find a similar correlation in healthy controls (**Figures 5A, B** **)**.

**Figure 6:**
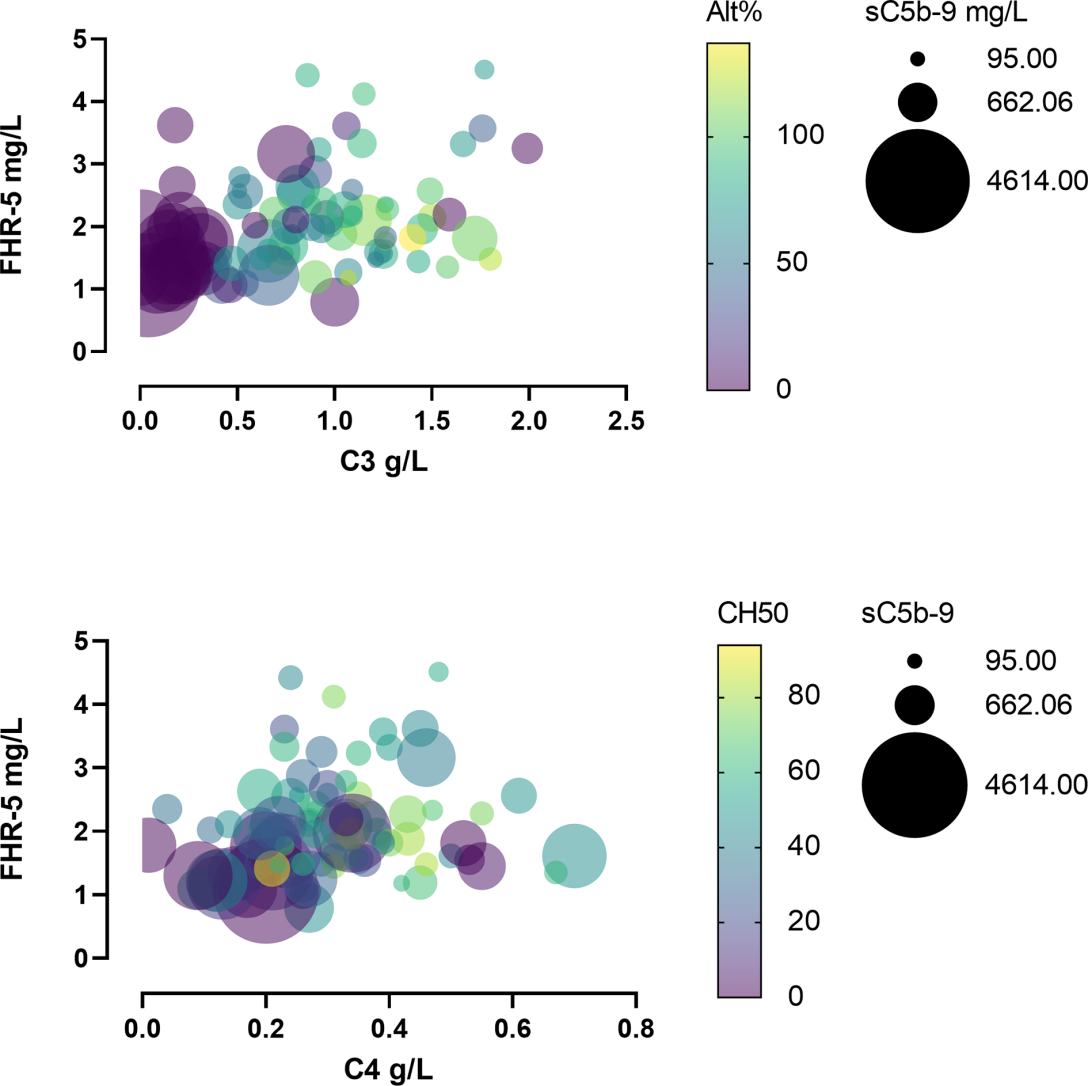
Bubble plot of patients’ various complement parameters and Factor H-related protein 5 (FHR-5) levels.

When we analyzed the patients’ C3, C4, sC5b-9, and FHR-5 levels together, it turned out that severe complement activation is accompanied by lower FHR-5 serum levels and hypocomplementemia (**Figure 6**).

### Prognostic Significance of FHR-5 Levels

**Figure 7:**
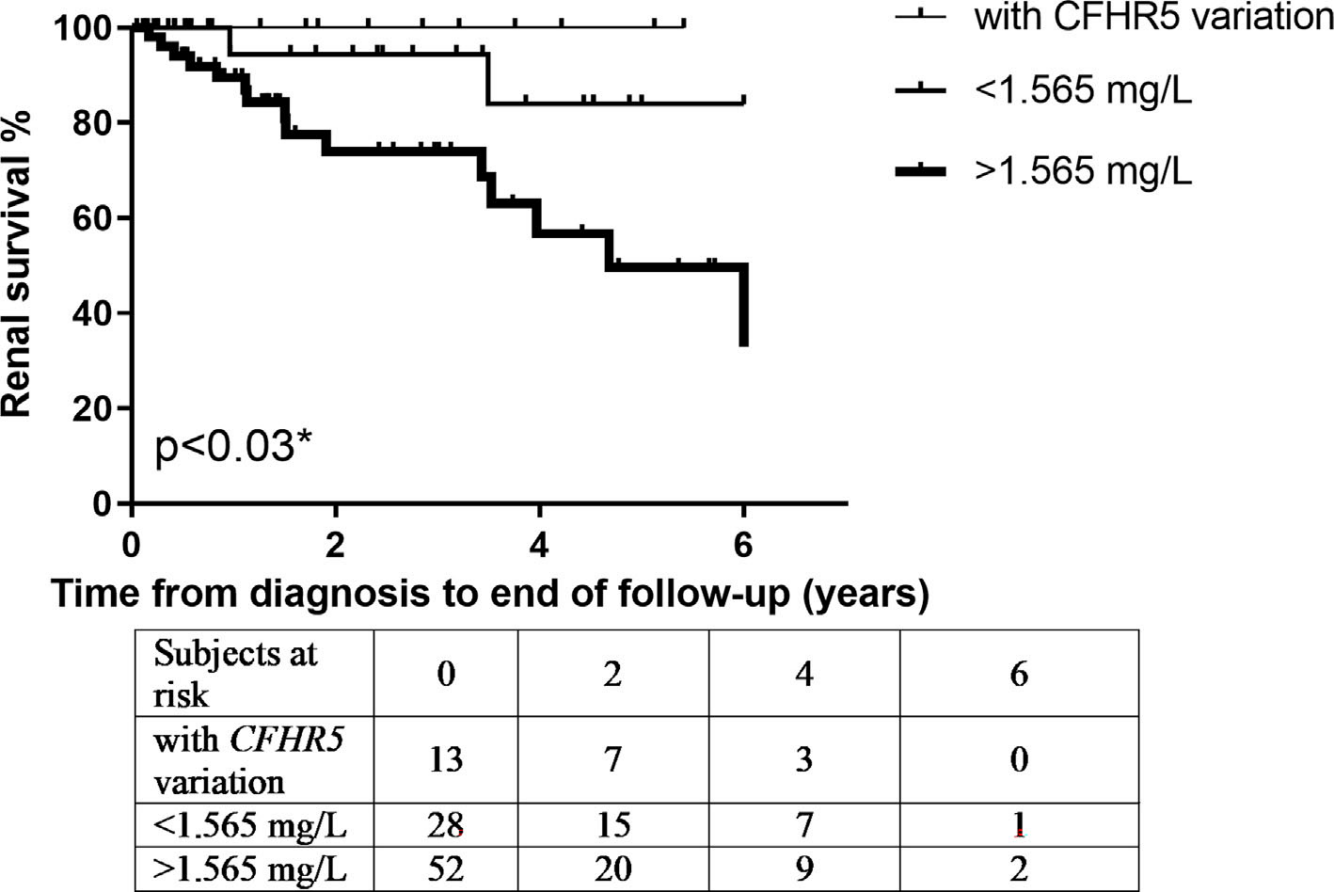
Patients’ renal survival according to their Factor H-related protein 5 (FHR-5) serum levels. * p-value was determined by log-rank test comparing patients with high and low FHR-5 serum levels.

**Figure 8:**
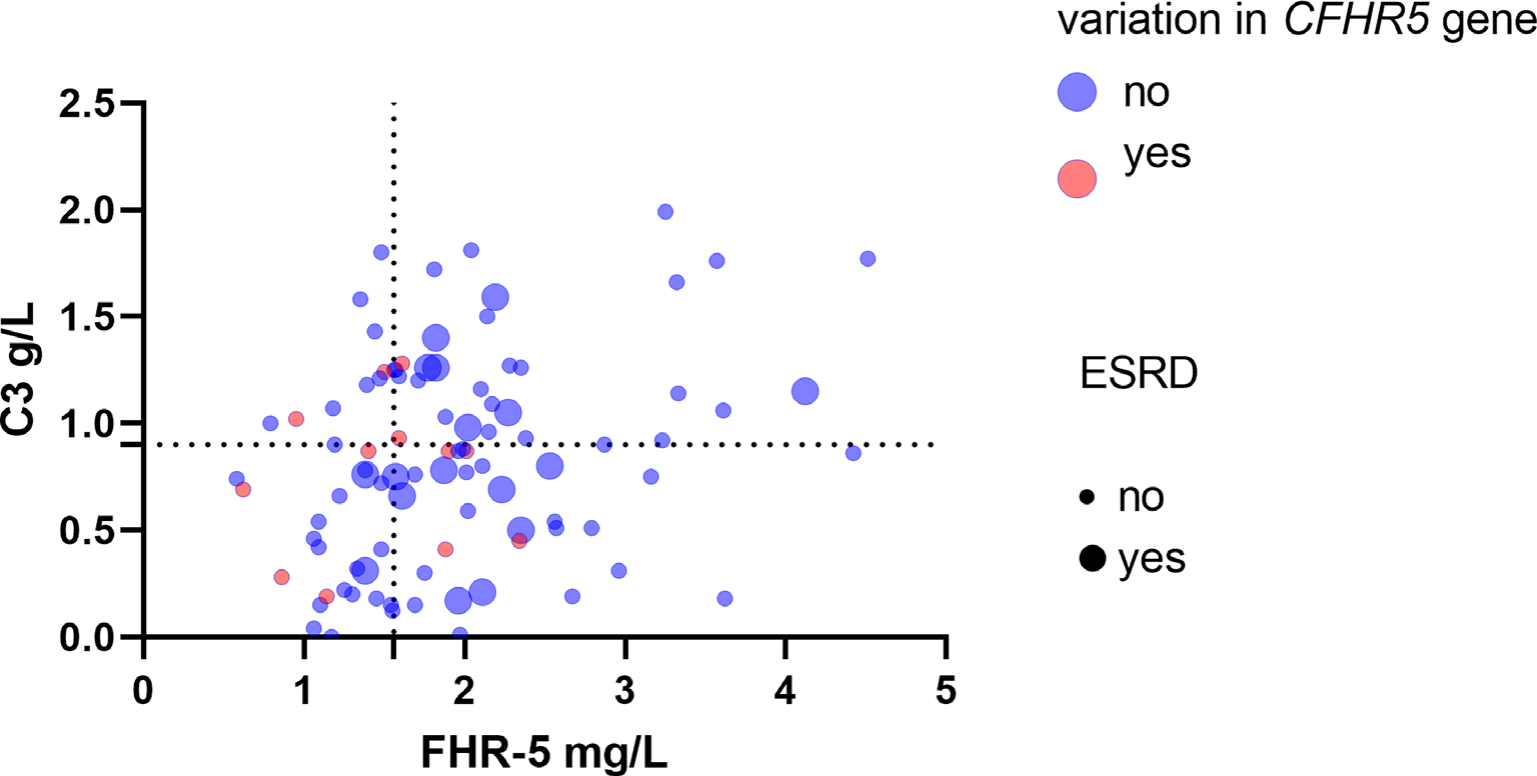
Patients’ Factor H-related protein 5 (FHR-5) and C3 levels along with the presence of *CFHR5* variations and end-stage renal disease (ESRD). Dotted lines indicate the threshold of high and low C3 (0.9 g/L) and FHR-5 (1.565 mg/L) levels. Rates of ESRD during follow-up - C3 <0.9 g/L, FHR-5 <1.56 mg/L: 0.032/event/patient/year, median (min–max) follow-up: 1.6 (0.11–6) - C3 <0.9 g/L, FHR-5 >1.56 mg/L: 0.11/event/patient/year, median (min–max) follow-up: 1.5(0.05–6) - C3 >0.9 g/L, FHR-5 <1.56 mg/L: 0/event/patient/year; median (min–max) follow-up: 1.57 (0.21–6) - C3 >0.9 g/L, FHR-5 >1.56 mg/L: 0.099 event/patient/year; median (min–max) follow-up: 1.5 (0.13–6).

Next, based on results of ROC analysis, patient groups with high or low baseline FHR-5 levels were formed (cutoff point 1.565 mg/L) to see if FHR-5 levels are associated with the development of ESRD during follow-up. Follow-up was successful for 103 patients (among whom 93 had genetic analysis) (median: 1.51 years; min–max: 0.05–6). Patient characteristics according to high (median: 2.16 mg/L; 1.87–2.85) or low (median: 1.34 mg/L; 1.12–1.46) FHR-5 serum levels are shown in **Table 4**. The 14 patients with *CFHR5* variations are presented as a separate group for reference (median: 1.6 years; min–max: 0.25–6). Seventeen out of the 93 patients (median: 1.53 years; min–max: 0.05–6 years) progressed or stayed in ESRD (**Table 5**) during follow-up. None of the patients with *CFHR5* variation(s) progressed to ESRD during follow-up (**Table 4**, **Figure 8**). Fifteen patients with high FHR-5 levels (ESRD rate: 0.38 event/patient/year; median follow-up: 4.5 years, min–max: 0.05–6) progressed to ESRD, whereas the same was observed only for two patients with low FHR-5 (ESRD rate: 0.04 event/patient/year; median follow-up: 1.98 years, min–max: 0.11–6) concentrations. Patients with higher FHR-5 levels had the worst renal survival when compared to patients with low FHR-5 concentrations (p = 0.034) or when the three groups were analyzed together (p = 0.016) (**Figure 7**).

**Table 4:** Presenting clinical and laboratory characteristics of patients with or without *CFHR5* variations, stratified according to FHR-5 serum levels.

	Patients with *CFHR5* variations*n = 13	Patients without *CFHR5* variation		p-value**
		serum FHR-5 <1.565 mg/L n = 28	serum FHR-5 >1.565 mg/L n = 52	
Sex % men	7 (53.8)	13 (46.4)	33 (63.4)	0.14
Age at diagnosis	17 (8–29)	16 (11–26)	31 (15–47)	0.012
Non-visible hematuria, present	7 (53.8)	19 (67.8)	32 (61.5)	0.57
Visible hematuria, present	3 (23)	5 (17.8)	10 (19.6)	0.77
Nephrotic syndrome, present	8 (61.5)	12 (42.8)	28 (54.9)	0.3
Renal impairment, present	6 (46.2)	8 (29.6)	23 (45)	**0.03**
Acute renal failure, present	0 (0)	2 (7.4)	7 (13.7)	0.48
Sclerosis on light microscopy (%)	0 (0–5.2)	6.7 (0–17.8)	14.2 (0–44.4)	0.17
Crescent on light microscopy (%)	0 (0–5.8)	0 (0–6)	0 (0–17.6)	0.21
ESRD during follow-up period, present	0 (0)	2 (7.1)	15 (28.8)	**0.02**
Serum C3, g/L	0.64 (0.24–0.78)	0.37 (0.21–0.98)	0.8 (0.3–1)	0.21
Serum C4, g/L	0.18 (0.10–0.25)	0.21 (0.13–0.26)	0.24 (0.2–0.35)	**0.02**
sC5b-9, ng/ml	534 (375–751)	517 (273–1277)	349 (244–574)	0.09
Decreased C3, present	9 (69.2)	19 (67.8)	25 (48)	0.08
Decreased C3 with normal C4, present	4 (30.8)	5 (17.8)	3 (5.7)	0.12
Elevated sC5b-9, present	11 (84.6)	19 (79.2)	36 (72)	0.58
Classical pathway activity, CH50/ml	37 (12–49)	35 (3.7–60.7)	45.5 (29–60)	0.17
Alternative pathway activity, %	27 (0.75-67)	8 (1–81)	61 (6–88)	0.1
Serum FHR-5, mg/L	1.54 (0.92–1.88)	1.34 (1.12–1.46)	2.16 (1.87–2.85)	**<0.0001**

Data presented are number (%) or median (interquartile range).

*Patients with CFHR5 variations are shown for reference.

^**^p-values were obtained by Mann–Whitney U test or χ^2^ test, comparing patients with serum FHR-5 <1.565 mg/L and patients with serum FHR-5 >1.565 mg/L.

ESRD, end-stage renal disease; FHR-5, Factor H-related protein 5.

p-values < 0.05 are shown in bold.

**Table 5:** Clinical characteristics of patients at the time of diagnosis with or without ESRD development during the follow-up period.

	Patients with ESRD during follow-up periodn = 17	Patients without ESRD during follow-up periodn = 75	p-value*
Sex % men	10 (58.8)	42 (56)	0.83
Age at diagnosis	40 (17–51)	18 (12–36)	0.035
Follow-up period (years)	1.6 (0.66–3.73)	1.5 (0.7–3.5)	0.83
Non-visible hematuria, present	13 (76.5)	44 (58.6)	0.17
Visible hematuria, present	3 (17.6)	15 (20)	0.82
Nephrotic syndrome, present	12 (70.6)	36 (48)	0.75
Renal impairment, present	10 (58.8)	27 (36)	0.08
Renal failure, present	5 (29.4)	4 (5.3)	**0.002**
Sclerosis on light microscopy (%)	27 (7–53)	1.6 (0–18.3)	0.004
Crescent on light microscopy (%)	0 (0–17.33)	0 (0–7.08)	0.78
Serum C3, g/L	0.78 (0.58–1.27)	0.87 (0.41–1.2)	0.8
Serum C4, g/L	0.24 (0.19–0.39)	0.28 (0.22–0.36)	0.71
sC5b-9, ng/ml	350 (234–615)	421 (272–725)	0.25
Decreased C3, present	10 (58.8)	42 (56)	0.8
Decreased C3 with normal C4, present	9 (52.9)	32 (42.6)	0.92
Elevated sC5b-9, present	10 (62.5)	55 (73.3)	0.23
Classical pathway activity, CH50/ml	48 (30.5–66.5)	43 (21–60)	0.21
Alternative pathway activity, %	76 (21–91)	38 (1–83)	0.08
Serum FHR-5, mg/L	1.96 (1.69–2.25)	1.69 (1.35–2.34)	0.18
Patients with *CFHR5* variations	0 (0)	13 (17.33)	0.06

Data presented are: number (%) or median (interquartile range).

*Group comparisons were made with Mann–Whitney U test or χ^2^ test.

ESRD, end-stage renal disease; FHR-5, Factor H-related protein 5.

p-values < 0.05 are shown in bold.

We also investigated whether any other complement parameter has any connection with renal survival. Patients with lower CP or AP activity and elevated sC5b-9 level at the time of diagnosis had better renal survival as well (**Supplementary Figure 2**).

We illustrated together patients’ FHR-5 and C3 levels, along with the presence of *CFHR5* variations and the outcome. In patients with lower C3 and FHR-5 levels (suggesting severe complement activation), ESRD occurred less frequently during the disease course (**Figure 8**).

### Distribution of FHR-5 Levels in Pathophysiological Clusters of Patients

**Figure 9:**
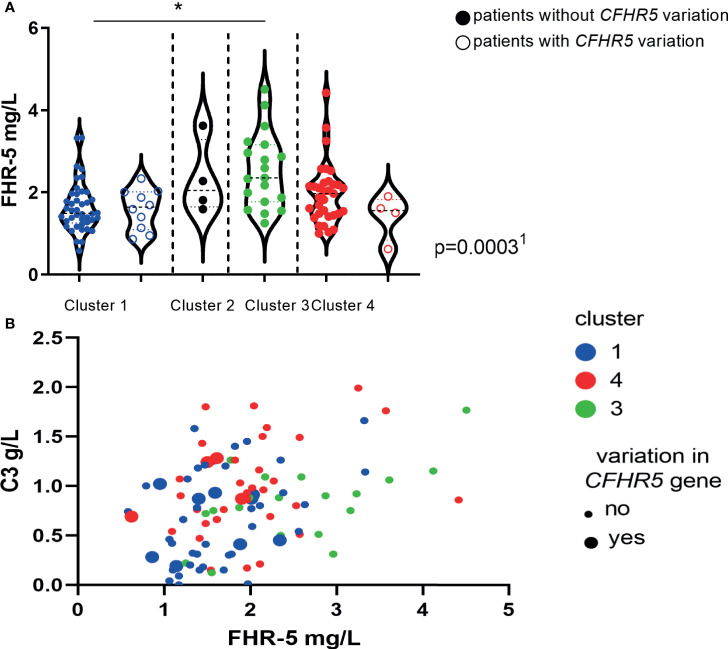
**(A)** Factor H-related protein 5 (FHR-5) protein levels in the previously described clusters of C3 glomerulopathy (C3G)/immune complex-mediated membranoproliferative glomerulonephritis (IC-MPGN) patients (19, 37). The clinically meaningful clusters were generated based on clinical, histological, genetic, and complement data of patients as described by Iatropoulos et al. (19). Each dot represents one patient. ^1^p-value was determined with ANOVA for patients without *CFHR5* variations comparing patients in clusters 1, 3, and 4. *p < 0.05 by Dunn’s posttest, for patients without *CFHR5* variations. **(B)** C3 and FHR-5 levels according to cluster membership and the presence of *CFHR5* variations.

We have also examined the distribution of serum FHR-5 levels across the previously described clusters (4, 19) and observed a clear association: patients in cluster 3 (median: 2.35 mg/L, IQR: 1.77–3.16) and cluster 4 (median: 1.96 mg/L, IQR: 1.48–2.23) had significantly higher FHR-5 levels when compared to cluster 1 (1.47 mg/L, 1.25–1.98, p < 0.05, Dunn’s *post-hoc* test, p = 0.0003, ANOVA; **Figure 9A**). The presence of patients with *CFHR5* variations was the highest in cluster 1 (10/49; 20.4%) when compared to the other clusters (4/58; 6.9%) (p = 0.047, χ^2^ test; **Figure 9A**), whereas development of ESRD during follow-up occurred less frequently among patients in cluster 1 (2/43; 4.8%) compared to patients in cluster 3 (5/17; 29.4%; p = 0.016) and cluster 4 (9/33; 27.3%; p = 0.007). Complement activation was clearly characteristic for cluster 1 when C3 and FHR-5 levels were analyzed together (**Figure 9B**).

When analyzing the data of patients’ treatment strategy at the time of diagnosis, no relevant differences were observed between the patients’ initial therapeutic options in the different clusters.

## Discussion

In this study, we have examined the FHR-5 serum levels and the *CFHR5* genetic variations in a large group of IC-MPGN/C3G patients and in healthy controls.

To the best of our knowledge, this is the first observational study describing FHR-5 levels together with the presence of *CFHR5* variations as well as clinical and laboratory data in a reasonably large group of IC-MPGN/C3G patients.

Similarly to C3, C4, CP, and AP activity, FHR-5 levels were lower in patients than those in healthy controls. Patients with low FHR-5 levels had superior renal survival compared to patients with higher FHR-5 levels, and this association was independent of *CFHR5* variation carrier status. Interestingly, FHR-5 levels and *CFHR5* variations showed a clear association with clusters of the patients: patients with hypocomplementemia, low FHR-5 levels, presence of *CFHR5* variations, and good renal outcome fall into cluster 1, whereas patients in clusters 3 and 4 had higher levels of FHR-5 with worse renal outcome.

A few genetic alterations of *CFHR5* were published to be associated with MPGN II/DDD by Abrera-Abeleda et al. (27), and later, a specific well-characterized *CFHR5* mutation was described by Gale et al. (28) as a pathogenic factor, where they showed that internal duplication of exons 2–3 of *CFHR5* leading to the expression of a mutant FHR-5 protein with duplicated SCR1 and 2 domains causes familial C3G termed CFHR5-nephropathy.

FHR-5 was reported to be colocalized in renal tissue together with other complement-containing immune deposits (20, 67), although the pathogenic role of this protein was not fully understood and it was hypothesized that it may have a physiological role in complement activation in the kidney, but large observational studies are missing.

In our IC-MPGN/C3G cohort, 12.6% of the patients carry *CFHR5* variations, representing eight different missense/frameshift variations, but their function is not entirely clear. There were no differences with regard to the patients’ clinical and laboratory characteristics except for FHR-5 level when we compared patients with or without *CFHR5* variations. The incidence of some *CFHR5* variations in our cohort was slightly elevated compared to those registered in worldwide databases; however, with regard to the low number of cases, no statistical comparison was made and no further conclusions can be drawn from these data.

The identified missense/frameshift *CFHR5* variations affect the SCR1–6 domains of the FHR-5 protein. FHR-5 was shown to form only homodimers in plasma (68), which is mediated *via* SCR1-SCR2 (23), that were affected by two different substitutions (K144N and V110A) in our cohort. These variations may influence the ligand binding [such as properdin (69)] or the dimerization ability of FHR-5, but functional studies are needed to confirm this hypothesis. We also identified two different frameshift mutations that are caused by the insertion of one or two adenine bases (E163Rfs*35; E163Kfs*10). Interestingly, in a previous case report, the E163Rfs*35 mutation occurred along with low FHR-5 concentration in one patient suffering from glomerulonephritis following streptococcal infection but not in unaffected carriers (36). In our cohort, the FHR-5 levels of these three patients with frameshift mutations were not markedly decreased in each case when analyzed by ELISA and WB methods. This could be explained by the fact that these patients are heterozygous and the intact allele may compensate for the loss-of-function allele.

The remaining two missense variations (G278S and R356H) affected the SCR4–6 domains that are partly responsible for the binding of C-reactive protein (22), heparin [SCR5-7 (22, 70)], laminin [SCR5-7 (70)], and necrotic human endothelial cells [SCR5-7 (70)] based on previous studies (and the binding of pentraxin-3 to SCR5-7 was also suggested) (71). Three patients carrying the G278S variation had variable levels of FHR-5, and both patients carrying the R356H variation had FHR-5 concentration below the median level observed in the controls. By analyzing recombinant proteins harboring these most frequent variations, we found that the FHR-5_G278S_ variant has decreased C3b-binding ability (**Figure 4**). Nevertheless, C3b is only one of the common FH/FHR ligands; the function of FHR-5 and the effect of mutations should be further investigated. In another perspective, interaction between FHR proteins and the extracellular matrix components can also modify the regulator activity of FHR proteins. The interactions between FHR-5 protein and the surface components such as glycosaminoglycans may also play a role in complement dysregulation (26). The role of variations in *CFHR5* in C3G, which is considered a polygenic disease, is not clear. Specific forms of FHR-5 protein can be disease-modifying in C3G, as seen in CFHR5 nephropathy, but the role of missense variations and frameshift mutations needs further investigation. In recent years, two studies performing *CFHR5* sequencing in aHUS patients (n = 54 and n = 65) were published that reported some novel *CFHR5* variations, including two alterations coding for K144N and R356H that were observed in our patients as well (31, 32). In a large American cohort (n = 104) of C3G patients, four heterozygous *CFHR5* variations were reported (including the G278S variation detected in three of our patients as well) without further comparison with serum levels and clinical data (37). On the other hand, a large study was reported including 500 IgAN patients (33) carrying several *CFHR5* polymorphisms, but none of these variations were observed in our patients. FHR-5 levels were higher in IgAN patients than in control subjects in several studies (34, 35), and higher FHR-5 concentrations were associated with the progression of IgAN (34).

Our study is the first that performed FHR-5 level measurement in a large IC-MPGN/C3G cohort along with the detection of the genetic background and clinical data. FHR-5 levels were significantly lower in patients with or without *CFHR5* variations compared to controls, which is in line with the observations of Vernon et al. (36) who found decreased FHR-5 levels in 23 patients with C3GN, with or without *CFHR5* alterations, compared to controls. Not only FHR-5 but other complement parameters were also lower in patients compared to controls, and FHR-5 level showed a clear correlation with complement levels, supporting the idea that increased complement activation and consumption may cause these differences.

FHR-5 levels showed a clear association with renal survival, as survival was better in IC-MGPN/C3G patients with lower FHR-5 levels than in patients with higher FHR-5 levels. This observation is similar to that obtained for IgAN patients (34). From another point of view, not only FHR-5 serum level but also the CP and AP activity and sC5b-9 level have connection with renal survival. It seems that higher complement activation and consumption at the time of diagnosis associated with better renal survival. The background is not clear; however, this result is in line with our previous observation (38) where patients with a higher complement activation in cluster 1 have better renal survival. It is possible that patients with more severe disease onset receive treatment more rapidly compared to patients with mild initial symptoms. These patients with latent disease progression may present in the health care unit when there are already chronic changes present in the kidney. Although the therapeutic strategies do not differ among the clusters, the time period between the disease onset and the initialization of therapy may be shorter in case of cluster 1. This is one of the limitations of our study, as there are no objective data available to examine this hypothesis more properly.

Our observations raise the possibility that plasma FHR-5 level has a role in C3G, but whether it takes part in the pathogenesis or it is a consequence of the disease course and complement activation is still unknown. We detected different *CFHR5* variations in our cohort; however, a clear molecular pathogenicity of the protein was not confirmed. As no statistical comparison between cases and controls was performed, we cannot exclude that certain *CFHR5* variations may have a disease risk-modifying role; however, our results suggest that these variations rather have an impact on the disease course of the carriers. Recently, a large-scale whole-genome sequencing study did not find a clear relationship between the identified rare variations and C3G; however, a strong association was identified between primary MPGN and a haplotype containing DQA1*05:01, DQB1*02:01, and DRB1*03:01. Of these, DQB1*02:01 and DRB1*03:01 are associated with different autoimmune diseases such as rheumatoid arthritis and membranous nephropathy. These genes are coding components of the MHCII molecule (found on the surface of antigen-presenting cells), which plays an important role in the adaptive immune response and in (auto)antibody production. These results raise the possibility that although genetic variations could have a disease-modifying effect, it is the aberrant adaptive immune mechanism, thus autoimmunity, that could be the key mechanism in the background of C3G (as shown by the high occurrence of autoantibodies) rather than the genetic abnormalities (72).

We have further analyzed whether *CFHR5* variations and serum FHR-5 concentrations are in connection with the recently described (19) and validated clinically meaningful clusters (4). IC-MPGN/C3G patients were clustered based on clinical, histological, complement, and genetic data, and a clear association with disease pathogenesis and renal survival was observed, supporting the relevance of the clusters, and our study found that FHR-5 levels were lower in cluster 1 along with higher prevalence of *CFHR5* variations. Cluster 1 was also characterized by younger age of onset, higher complement activation with higher prevalence of complement autoantibodies, and better renal survival. On the contrary, worst renal survival was observed in clusters 3 and 4 in our study, and patients in clusters 3 and 4 had higher FHR-5 levels. The extent of CP/AP complement activation in cluster 1 was higher compared not only to the other clusters [as described in our previous study (38)] but also to healthy controls (as shown by the measured low levels). Overall, it was shown that patients with MPGN have higher extent of *in vivo* complement activation compared to healthy controls.

In conclusion, our study is the first to report observational data on serum FHR-5 levels at disease presentation and *CFHR5* variations in a large group of IC-MPGN/C3G patients, where we observed that 14 patients (12.6%) were carriers of eight different *CFHR5* variations (**Table 3**). Low serum FHR-5 concentration at presentation was associated with decreased incidence of ESRD during follow-up. Low serum FHR-5 levels showed clear association with signs of hypocomplementemia and clinically meaningful groups (clusters) of patients. According to our results, it seems that lower FHR-5 levels detected in patients are part of the severe complement activation leading to hypocomplementemia. Further studies, including detailed *in vitro* characterization of the functional effects of the identified *CFHR5* variations, are necessary to better understand the role of FHR-5 in the pathogenesis of complement-mediated kidney diseases. These investigations are in progress in our laboratories.

## Data Availability Statement

The raw data supporting the conclusions of this article will be made available by the authors, without undue reservation.

## Ethics Statement

The studies involving human participants were reviewed and approved by Medical Research Council of the Ministry of Human Capacities in Hungary (approval’s number:55381-1/2015/EKU) and the Institutional Review Board of the Semmelweis University, Budapest. Written informed consent to participate in this study was provided by the participants’ legal guardian/next of kin.

## Author Contributions

ZP, DCs, ÁS, NG, and MJ contributed to the study design. DCs, NV, EdS, NG, MC, BU, and AI contributed to the experiments. ZP, DCs, ÁS, NV, EdS, NG, BU, and AI contributed to the data analysis. CA, ASc, MG, GS-P, DB, JB, AD, DCe, SF, HF, ÁHari, ÁHart, AH, TM, KR, KA, JH, DJ, MSi, ErS, CB, VJ, KKe, GR, ASz, NKl, KKo, NKo, MK, ML, ACL, AM, RRu, TKL, EM, MM, AP, TSt, LP, MR, GM, RRy, JR, MSa, TSe, JZ, ES, TSz, AC, SS, MT, KG, AT, IV, MW, TZ, GZ, ZP, DCs, ÁS, and NG contributed to data collection. All authors contributed to the article and approved the submitted version.

## Funding

This work was supported by a grant of the Premium Postdoctoral Fellowship Program of the Hungarian Academy of Sciences (PPD2018-016/2018) to DCs. The research was financed by the Higher Education Institutional Excellence Program of the Ministry of Human Capacities in Hungary within the framework of the molecular biology thematic program of Semmelweis University and “Befektetés a jövőbe” (2020-1-1-6-JÖVŐ-2021-00013) by NKFIH to ZP. NG received financial support from the EFOP-3.6.3-VEKOP-16-2017-00009 grant. MJ was supported by the Kidneeds Foundation (Iowa, USA), the Hungarian Academy of Sciences (nr. 0106307), and the National Research, Development and Innovation Fund (OTKA, grant K 125219), and the Institutional Excellence Program to ELTE (NKFIH-1157/8/2019, D11206).

## Conflict of Interest

Author VJ was employed by company Medimpax.

The remaining author declares that the research was conducted in the absence of any commercial or financial relationships that could be construed as a potential conflict of interest.

## Publisher’s Note

All claims expressed in this article are solely those of the authors and do not necessarily represent those of their affiliated organizations, or those of the publisher, the editors and the reviewers. Any product that may be evaluated in this article, or claim that may be made by its manufacturer, is not guaranteed or endorsed by the publisher.

## Abbreviations

wt, wild-type; het, heterozygous; C3G, C3 glomerulopathy; DDD, dense deposit disease; FHR-5, Factor H-related protein 5; IC-MPGN, immune complex-mediated membranoproliferative glomerulonephritis.
