# Supplementary figures and images for: FHR-5 Serum Levels and CFHR5 Genetic Variations in Patients With Immune Complex-Mediated Membranoproliferative Glomerulonephritis and C3-Glomerulopathy
Source: Front Immunol. 2021 Sep 10;12:720183. doi: 10.3389/fimmu.2021.720183 (PMC8461307; doi:10.3389/fimmu.2021.720183)

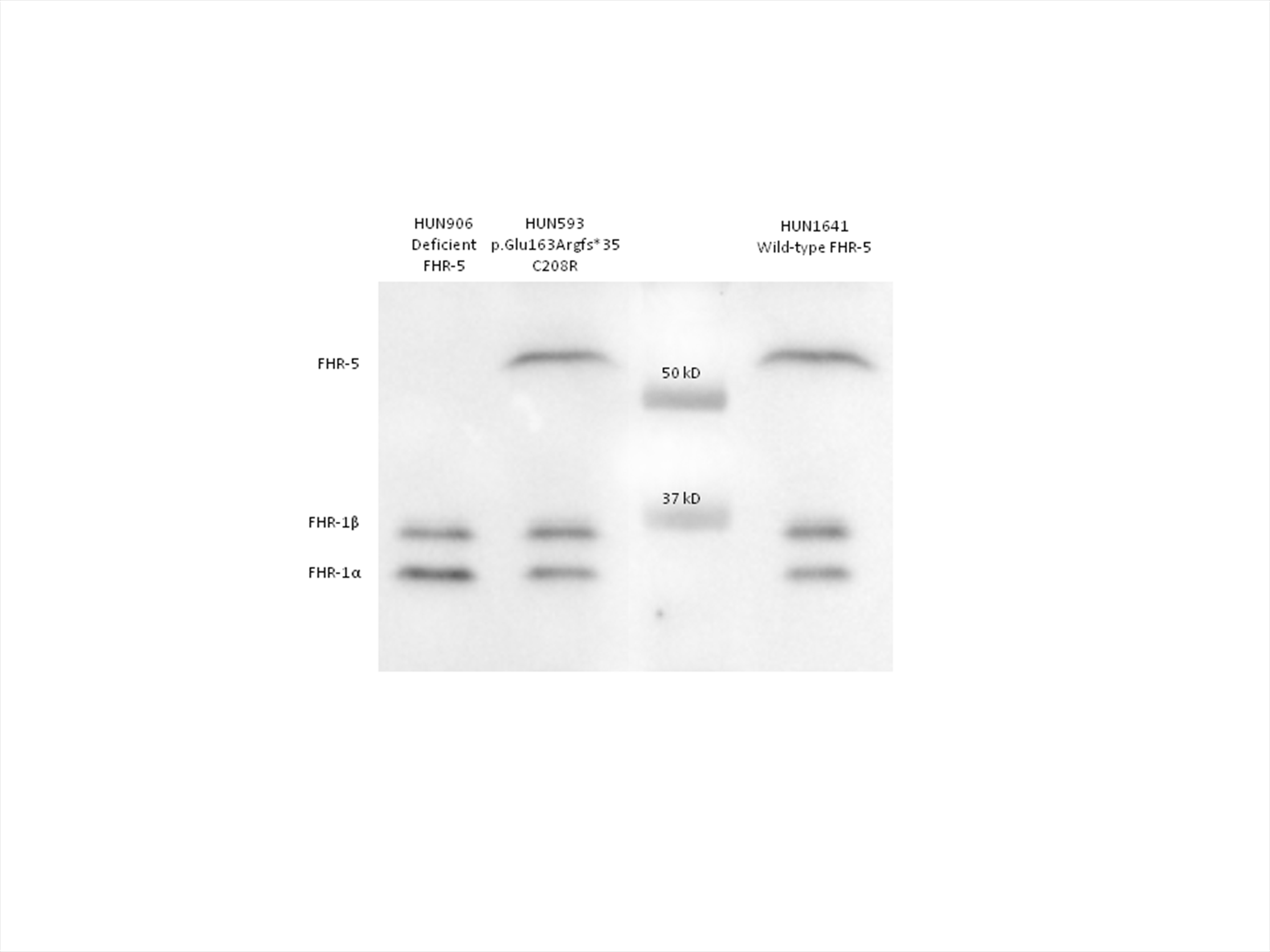

Supplement: Supplementary file 1 [file Image_1.tif]

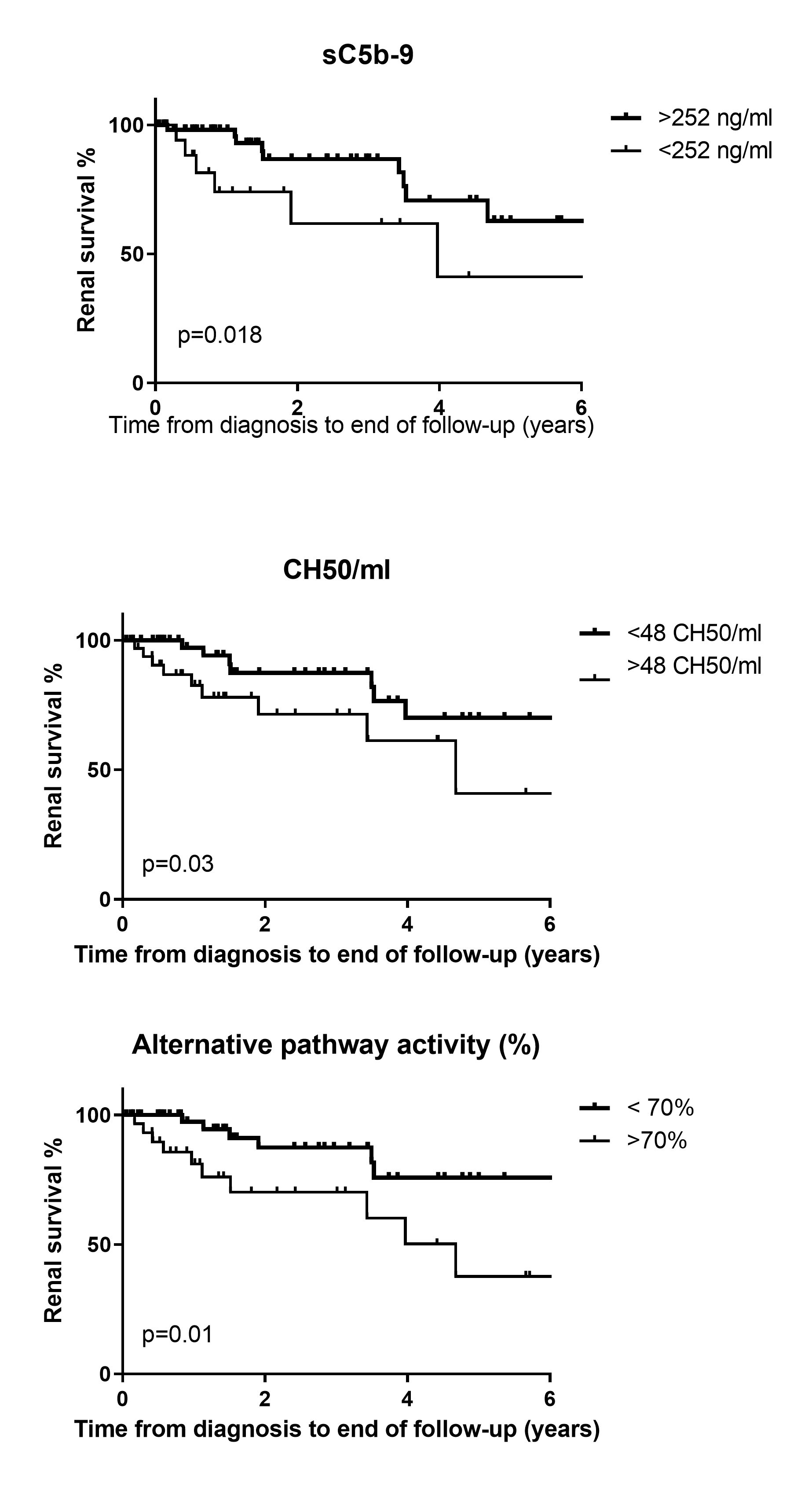

Supplement: Supplementary file 2 [file Image_2.tif]
