# Supplementary material for: FHR-5 Serum Levels and CFHR5 Genetic Variations in Patients With Immune Complex-Mediated Membranoproliferative Glomerulonephritis and C3-Glomerulopathy
Source: Front Immunol. 2021 Sep 10;12:720183. doi: 10.3389/fimmu.2021.720183 (PMC8461307; doi:10.3389/fimmu.2021.720183)
Supplement: Supplementary file 3 [file Table_1.docx]

**Supplementary Table 1.** Minor allele frequencies (%) of the identified *CFHR5* variations.

| **Amino acid change in FHR-5** | ***CFHR5* cDNA position** | **rs number** | **Minor allele frequencies (%)** | | | |
| --- | --- | --- | --- | --- | --- | --- |
|  |  |  | **1000 Genomes Project** (European) | **gnomAD** (non-Finnish European) | **EVS** (European American) | **Our cohort** |
| **P46S** | c.136C>T | rs12097550 | 0.80 | 0.63 | 0.79 | 0.90 |
| **V110A** | c.329T>C | [rs140691305](http://www.ncbi.nlm.nih.gov/projects/SNP/snp_ref.cgi?searchType=adhoc_search&type=rs&rs=rs140691305) | 0.00 | 0.02 | 0.01 | 1.80 |
| **K144N** | c.432A>T | [rs181511327](http://www.ncbi.nlm.nih.gov/projects/SNP/snp_ref.cgi?searchType=adhoc_search&type=rs&rs=rs181511327) | 0.20 | 0.10 | 0.10 | 0.45 |
| **E163Kfs*10** | c.479_480insAA | rs565457964 | 0.89 | 0.61 | 0.39 | 0.45 |
| **E163Rfs*35** | c.479_480insA | rs565457964 | 0.20 | 0.31 | 0.34 | 0.90 |
| **C208R** | c.622T>C | [rs41299613](http://www.ncbi.nlm.nih.gov/projects/SNP/snp_ref.cgi?searchType=adhoc_search&type=rs&rs=rs41299613) | 0.10 | 0.21 | 0.22 | 0.90 |
| **G278S** | c.832G>A | [rs139017763](http://www.ncbi.nlm.nih.gov/projects/SNP/snp_ref.cgi?searchType=adhoc_search&type=rs&rs=rs139017763) | 1.39 | 0.98 | 0.88 | 1.35 |
| **R356H** | c.1067G>A | [rs35662416](http://www.ncbi.nlm.nih.gov/projects/SNP/snp_ref.cgi?searchType=adhoc_search&type=rs&rs=rs35662416) | 3.28 | 2.45 | 2.82 | 0.90 |

**Supplementary Table 2**. *In silico* predictions for the identified *CFHR5* variations

| **Identified *CFHR5* variation** | **Mutation Taster** | **PROVEAN** | **SIFT** | **Polyphen-2**  **(performed by the HumDiv model)** |
| --- | --- | --- | --- | --- |
| **P46S** | Polymorphism  (Probability: 0.99) | Neutral  (Score: -0.49) | Tolerated  (Score: 0.37) | Benign  (Score: 0.044, sensitivity: 0.94; specificity: 0.83) |
| **V110A** | Polymorphism  (Probability: 0.99) | Deleterious  (Score: -3.03) | Tolerated  (Score: 0.16) | Possibly damaging  (Score: 0.877, sensitivity: 0.83; specificity: 0.94) |
| **K144N** | Polymorphism  (Probability: 0.99) | Neutral  (Score: -0.36) | Tolerated  (Score: 0.17) | Benign  (Score: 0.058, sensitivity: 0.94; specificity: 0.84) |
| **E163Kfs*10** | Disease causing  (Probability: 1.00) | NA | NA | NA |
| **E163Rfs*35** | Disease causing  (Probability: 1.00) | NA | NA | NA |
| **C208R** | Polymorphism  (Probability: 0.98) | Deleterious  (Score: -11.56) | Damaging  (Score: 0.0) | Probably damaging  (Score: 1.00, sensitivity: 0.00; specificity: 1.00) |
| **G278S** | Polymorphism  (Probability: 0.99) | Deleterious (Score: -5.26) | Tolerated  (Score: 0.07) | Probably damaging  (Score: 0.99, sensitivity: 0.14; specificity: 0.99) |
| **R356H** | Polymorphism  (Probability: 0.99) | Neutral  (Score: -0.33) | Tolerated  (Score: 0.19) | Possibly damaging  (Score: 0.820, sensitivity: 0.84; specificity: 0.93) |

**Supplementary Figure Legends**

**Supplementary Figure 1.** Western blot of the patient (HUN593) carrying two mutations in *CFHR5*, by using polyclonal anti-FHR-5 antibody. Samples of patient HUN906 (having complete deficiency of FHR-5) and HUN1641 (wild-type FHR-5) are shown for reference.

**Supplementary Figure 2.** Patients’ renal survival according to their classical-, alternative pathway activity and sC5b-9 levels

*p-value was determined by log-rank test comparing patients with high and low FHR-5 serum levels
